# Design, synthesis, and analysis of antiproliferative and apoptosis-inducing activities of nitrile derivatives containing a benzofuran scaffold: EGFR inhibition assay and molecular modelling study

**DOI:** 10.1080/14756366.2021.1946044

**Published:** 2021-07-06

**Authors:** Salma Fares, Khalid B. Selim, Fatma E. Goda, Magda A. A. El-Sayed, Nawaf A. AlSaif, Mohamed M. Hefnawy, Alaa A.-M. Abdel-Aziz, Adel S. El-Azab

**Affiliations:** a Faculty of Pharmacy, Department of Pharmaceutical Organic Chemistry, Mansoura University, Mansoura, Egypt; b Faculty of Pharmacy, Department of Pharmaceutical Chemistry, Delta University for Science and Technology, Gamasa City, Egypt; c Department of Pharmaceutical Chemistry, Horus University, New Dammeitta, Egypt; d Department of Pharmaceutical Chemistry, College of Pharmacy, King Saud University, Riyadh, Saudi Arabia

**Keywords:** Benzofuran, antiproliferative activity, EGFR TK, cell cycle analysis, molecular modelling

## Abstract

New cyanobenzofurans derivatives **2–12** were synthesised, and their antiproliferative activity was examined compared to doxorubicin and Afatinib (IC_50_ = 4.17–8.87 and 5.5–11.2 µM, respectively). Compounds **2** and **8** exhibited broad-spectrum activity against HePG2 (IC_50_ = 16.08–23.67 µM), HCT-116 (IC_50_ = 8.81–13.85 µM), and MCF-7 (IC_50_ = 8.36–17.28 µM) cell lines. Compounds **2**, **3**, **8**, **10**, and **11** were tested as EGFR-TK inhibitors to demonstrate their possible anti-tumour mechanism compared to gefitinib (IC_50_ **=** 0.90 µM). Compounds **2**, **3**, **10**, and **11** displayed significant EGFR TK inhibitory activity with IC_50_ of 0.81–1.12 µM. Compounds **3** and **11** induced apoptosis at the Pre-G phase and cell cycle arrest at the G2/M phase. They also increased the level of caspase-3 by 5.7- and 7.3-fold, respectively. The molecular docking analysis of compounds **2**, **3**, **10**, and **11** indicated that they could bind to the active site of EGFR TK.

## 1. Introduction

**Figure 1.**
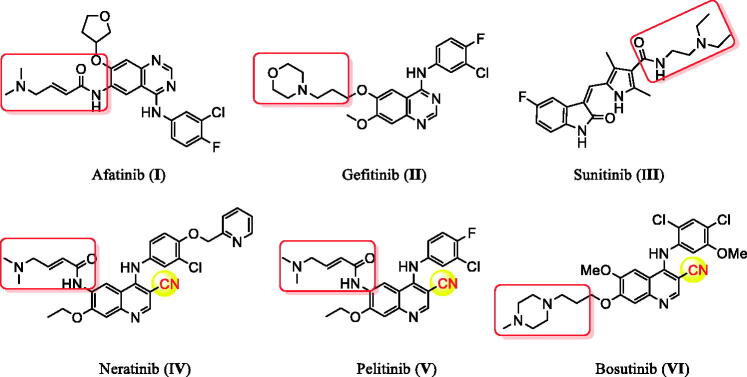
The reported anti-tumour agents with inhibitory activity against EGFR.

**Figure 2.**
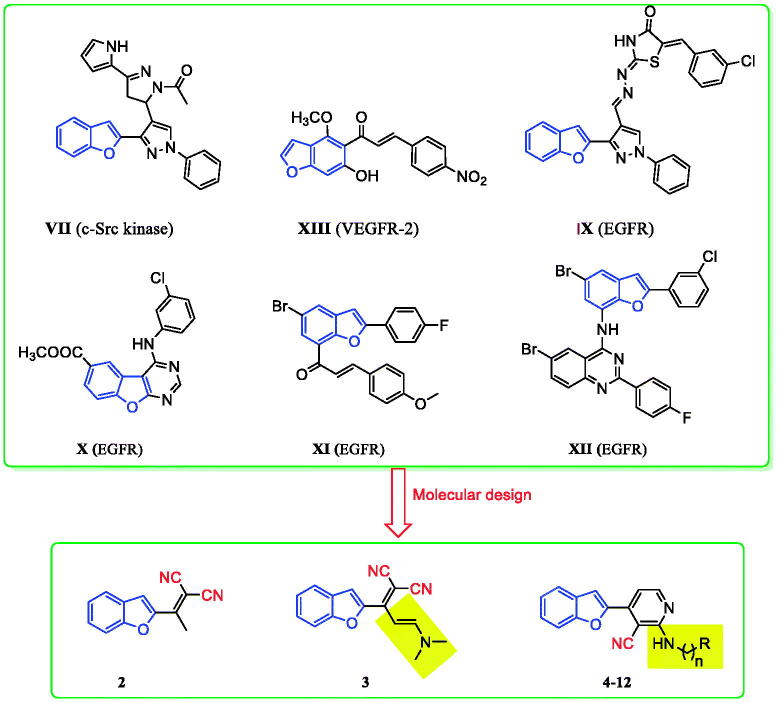
Previously reported benzofuran derivatives **VII–XII** with anti-tumour and kinase inhibition activities as well as compounds **2–12** designed herein.

Cancer remains among the leading causes of death worldwide owing to the resistance of cancer cells to the existing anti-tumour agents^1–3^. Treatment of cancer is a significant challenge for medicinal chemists due to the pressing need for novel and effective anti-cancer drugs^4–10^. Moreover, receptor tyrosine kinases (RTK) play crucial roles in activating signal transduction pathways in the cell, resulting in cell division, differentiation, and activation of regulatory mechanisms^11^^,^^12^. Epidermal growth factor receptor tyrosine kinase (EGFR-TK) is a tyrosine kinase receptor of the ErbB family. It regulates numerous biological processes, including cell motility, adhesion, regulation, angiogenesis, apoptosis, and metastasis^13–19^. Notably, overexpression of these receptors is found in various cancer cells (e.g. colon, ovarian, prostate, and breast cancer cells)^17–20^. Hence, simultaneous inhibition of EGFR is expected to provide superior efficacy to single receptor targeting, making EGFR a critical target for the design and development of anti-tumour agents^15^^,^^21–26^. In recent years, afatinib (**I**), and gefitinib (**II**), have been reported as effective EGFR inhibitors for the treatment of several cancer types (Figure 1)^27–32^. It has been found that compounds are possessing a nitrile moiety exhibit exciting and diverse biological activities^33^^,^^34^. For instance, neratinib (I**V**), pelitinib (**V**), and bosutinib (**VI**) are tyrosine kinase inhibitors incorporating nitrile groups. They have been shown to be effective in the treatment of breast cancer, solid tumours, and chronic myelogenous leukaemia, respectively (Figure 1)^35–37^. Intriguingly, the benzofuran core is one of the essential oxygen-containing scaffolds. Valuable therapeutic agents can be obtained by integrating suitable pharmacophores on the benzofuran moiety^38^^,^^39^. Recent studies indicated that benzofuran derivatives possessed anti-tumour activity^39–41^. In addition, benzofuran-containing compounds have been demonstrated to be kinase inhibitors. For instance, compound **VII** exhibited good inhibitory activity against c-Src, while chalcone-benzofuran **XIII** was a strong inhibitor of vascular endothelial growth factor receptor 2 (VEGR-2) (Figure 2)^42^^,^^43^. Recently, compounds incorporating a benzofuran core, such as derivatives **IX**, **X**, and **XI**, displayed significant EGFR TK inhibitory activity compared to the reference drug erlotinib. Compounds **IX**, **X**, and **XI** could effectively induce apoptosis (Figure 2)^26^^,^^44^^,^^45^. Furthermore, hybridisation of benzofuran with 4-aminoquinoline afforded compound **XII**, which showed inhibitory activity against EGFR (Figure 2)^46^.

Considering the above results, in this work, a series of benzofuran scaffolds **2–12** (Figure 2) was synthesised based on bioisosteric modifications of compounds shown in Figures 1 and 2. In the prepared derivatives, the benzofuran core was linked with alkylnitrile or nicotinonitrile moieties. The anti-cancer activity of the designed compounds was analysed using a 3-(4,5-dimethylthiazol-2-yl)-2,5-diphenyltetrazolium bromide (MTT) assay. The most active compounds were also evaluated against the target EGFR TK. Moreover, the induction of apoptosis and the effects of the most active derivatives on the caspase-3 level were assessed using a flow cytometry technique. The cell cycle activity was also detected for the most potent compounds to determine the possible cell cycle stage at which the new derivatives could suppress the growth of cancer cells. Lastly, molecular modelling was conducted to explore the plausible binding modes of the most promising derivatives in the binding site of EGFR.

## 2. Results and discussion

### 2.1. Chemistry

**Scheme 1.**
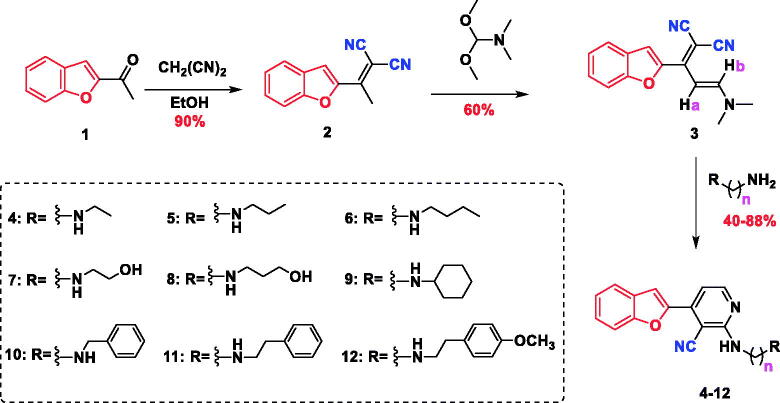
Synthetic route for the preparation of cyanobenzofuran derivatives **2–12**.

The synthetic pathway adopted to prepare the novel series of benzofuran-incorporating nitrile derivatives is shown in Scheme 1. Knoevenagel condensation of 2-acetyl benzofuran (**1**) in an ethanolic solution of malononitrile afforded 2-(1-(benzofuran-2-yl)ethylidene)malononitrile (**2**), which reacted with *N*,*N*-dimethylformamide dimethylacetal (DMFDMA) to give (*E*)-2-(1-(benzofuran-2-yl)-3-(dimethylamino)allylidene)malononitrile (**3**). The subsequent condensation reaction with different primary amines yielded 4-(benzofuran-2-yl)-2-(substituted)-nicotinonitriles **4–12**. The structure of compound **2** was confirmed by infra-red (IR) spectroscopy, which showed absorption bands at 2222 cm^−1^ (CN) and 1572 cm^−1^ (C=C). Additionally, the disappearance of a band at 1680 cm^−1^ was detected (C=O). The ^13^C nuclear magnetic resonance (NMR) spectrum revealed the disappearance of carbon signals at 186 ppm (C=O) as well as the appearance of two peaks at 113.24 and 113.60 ppm, which were attributed to two nitrile groups. Moreover, the presence of the methyl group of the ethylidene moiety (CH_3_–C=C) was confirmed by the singlet peaks at 2.63 and 19.38 ppm in the ^1^H NMR and ^13^C NMR spectra, respectively. The structure of compound **2** was verified by elemental analysis and mass spectrometry, which showed a molecular ion peak (M^+^) at *m/z* 208. The NMR spectrum of compound **3** was characterised by the absence of the methyl signal of the ethylidene moiety (CH_3_–C=C) at 2.63 and 19.38 ppm. In addition, two singlet peaks corresponding to the dimethylamino group (N(CH_3_)_2_) were observed at 3.09 and 3.23 ppm in the ^1^H NMR spectrum as well as at 37.86 and 45.77 ppm in the ^13^C NMR spectrum. The structures of compounds **4–12** were confirmed by IR, ^1^H NMR, ^13^C NMR, and mass spectrometry data. The IR spectra showed absorption signals at 3343–3370 and 2209–2219 cm^−1^ due to the presence of (NH) and (CN) groups, respectively. Moreover, the peaks corresponding to the dimethylamino group (N(CH_3_)_2_) at 3.09 and 3.23 ppm in the ^1^H NMR spectra as well as at 37.86 and 45.77 ppm in the ^13^C NMR spectra of compounds **4**–**12** disappeared. However, the signals ascribed to the (NH) group were detected at 6.66–7.18 ppm. The presence of the aliphatic residue was confirmed by the peaks at 0.90–4.56 and 13.49–59.28 ppm in the ^1^H NMR and ^13^C NMR spectra, respectively.

### 2.2. Biological screening

#### 2.2.1. *In vitro* antiproliferative activity and structure activity relationship (SAR)

**Figure 3.**
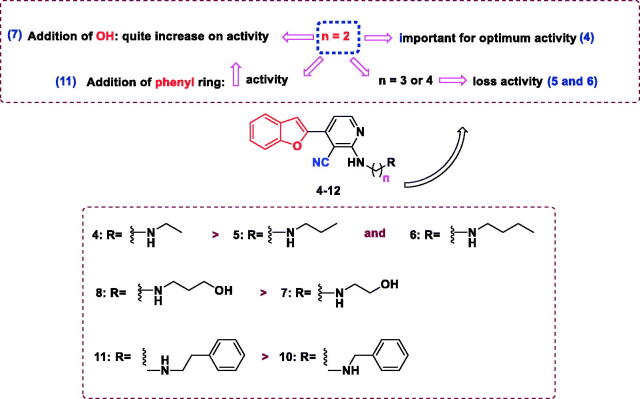
Structure activity relationship of benzofuran–nicotinonitrile derivatives as anti-cancer agents.

The antiproliferative activity of the newly synthesised compounds **2–12** against five human cancer cell lines, including hepatocellular carcinoma (HePG2), colorectal carcinoma (HCT-116), human breast adenocarcinoma (MCF-7), human prostate carcinoma (PC3), cervical carcinoma (HeLa), and normal cell (WI38) was evaluated by an MTT assay employing a previously described procedure^47^^,^^48^. Doxorubicin (DOX) and Afatinib were used as positive control. The antiproliferative activity of the tested compounds is summarised in Table 1 and Figure 3. DOX and Afatinib exhibited IC_50_ values of (4.50 and 5.53 µM), (5.23 and 11.23 µM), (4.17 and 7.23 µM), (8.87 and 7.6 3 µM), and (5.57 and 6.3 µM) against HePG2, HCT-116, and MCF-7, PC3, and HeLa cells, respectively. Compound **2**, which possessed two nitrile groups, showed strong antiproliferative activity, with IC_50_ values of 8.81 and 8.36 µM against HCT-116, and MCF-7 cell lines, respectively. However, **2** showed moderate activity against HePG2 with an IC_50_ value of 16.08 µM, and weak activity against PC3 and HeLa cell lines, with IC_50_ values of 26.82 and 39.03 µM, respectively. Enaminonitrile **3** displayed strong antiproliferative activity against the HCT-116 cell line (IC_50_ of 10.84 µM) and weak activity against the other tested cell lines. The benzofuran–nicotinonitrile derivatives **4–12** bearing secondary amine side chains exhibited varying antiproliferative activity. Derivative **4** with an ethylamine fragment showed strong antiproliferative activity against the PC3 and good activity against HeLa cell lines, with IC_50_ values of 14.27 and 21.10 µM, respectively. Nonetheless, compound **4** only weak activities were observed against HePG2 and MCF-7 cell lines (IC_50_ values of 58.75 and 55.04 µM, respectively). Notably, elongation of the ethyl chain (e.g. compound **4**, IC_50_ = 14.27–58.75 µM) to a propyl (e.g. derivative **5**) or a butyl moiety (e.g. compound **6**) led to decreased antiproliferative activity against HePG2, MCF-7, PC3, and HeLa cell lines, with IC_50_ values in the range of 75.19–100.0 µM. In contrast, derivative **6** showed better activity against the HCT-116 cell line than **4** and **5** with IC_50_ values of 56.36, 72.4, and 94.14 µM, respectively. The introduction of a hydroxyl group in compound **4** gave derivative **7**. It increased the antiproliferative activity against HePG2, HCT-116, and MCF-7 cell lines, with IC_50_ values of (58.75, 72.40, and 55.04 µM) (53.43, 38.64, and 40.83 µM), respectively. Conversely, the PC3 and HeLa cell lines were less sensitive to compound **7** than derivative **4** with IC_50_ (70.10 and 73.29 µM) and (14.27 and 21.10 µM). Intriguingly, introducing a hydroxyl group in compound 5 afforded derivative 8 and led to increased antiproliferative activity against all tested cell lines (IC_50_ range of 75.19 to >100.0 µM and 13.85 to 58.76 µM, respectively).

**Table 1.** *In vitro* antiproliferative activities (IC_50_, µM)^a,b^ of the synthesised compounds **2–12**.

Compound	*In vitr*o cytotoxicity (µM)					WI38^c^
	HePG2	HCT-116	MCF-7	PC3	HeLa	
**2**	16.08 ± 1.4	8.81 ± 0.7	8.36 ± 0.9	26.82 ± 2.2	39.03 ± 2.4	86.48 ± 4.7
**3**	40.02 ± 2.5	10.84 ± 1.0	27.36 ± 1.8	44.50 ± 3.5	68.98 ± 3.9	63.1 ± 3.43
**4**	58.75 ± 3.3	72.40 ± 3.8	55.04 ± 3.2	14.27 ± 1.3	21.10 ± 1.6	nt
**5**	83.56 ± 4.4	94.14 ± 5.1	>100	94.60 ± 4.9	75.19 ± 4.8	nt
**6**	79.04 ± 4.2	56.36 ± 3.2	81.58 ± 4.0	>100	91.90 ± 5.1	204 ± 11.1
**7**	53.43 ± 2.9	38.63 ± 2.0	40.83 ± 2.5	70.10 ± 3.8	73.29 ± 4.2	59.49 ± 3.23
**8**	23.67 ± 1.8	13.85 ± 1.3	17.28 ± 1.4	36.69 ± 2.8	58.76 ± 3.7	151.4 ± 8.22
**9**	44.70 ± 2.7	29.52 ± 1.8	35.17 ± 2.2	51.06 ± 3.6	73.59 ± 4.1	nt
**10**	34.32 ± 2.1	27.49 ± 1.5	19.69 ± 1.6	30.90 ± 2.6	46.17 ± 3.0	nt
**11**	20.43 ± 1.6	46.13 ± 2.7	14.55 ± 1.3	18.75 ± 1.5	32.15 ± 1.9	94.49 ± 5.13
**12**	70.46 ± 3.8	49.72 ± 2.9	61.53 ± 3.5	93.48 ± 4.4	85.44 ± 4.7	76.26 ± 4.14
**DOX**	4.50 ± 0.2	5.23 ± 0.3	4.17 ± 0.2	8.87 ± 0.6	5.57 ± 0.4	55.29 ± 3.0
**Afatinib**	5.5 ± 0.24	11.2 ± 1.11	7.2 ± 0.50	7.6 ± 0.60	6.3 ± 0.69	nt

^a^IC_50_ values for each cell line are the compound concentration that inhibits 50% of the cell growth measured by MTT assay. ^b^Each value was reproduced in triplicate. ^c^Non tumour normal cell.

Similarly, compared with derivative **7**, compound **8** exhibited a drastic increase in the antiproliferative activity against all evaluated cell lines (IC_50_ range of 38.64–73.29 µM and 13.85–58.76 µM, respectively). Replacement of the ethyl moiety in compound **4** with a cyclohexyl fragment (compound **9**) increased the antiproliferative activity against HePG2, HCT-116, and MCF-7 cell lines with IC_50_ values of (58.75, 72.40, and 55.04 µM) and 44.70, 29.52 and 35.17 µM), respectively. Furthermore, PC3 and HeLa cell lines were more susceptible to derivative **4** than compound **9** IC_50_ values of (14.27 and 21.0 µM) and 51.06–73.59 µM), respectively. Moreover, benzylamine derivative **10** comparatively inhibited the growth of MCF-7 cells (IC_50_ value of 19.69 µM) and showed weak activity against the other tested cell lines. It is noteworthy that the replacement of the benzyl moiety in compound **10** with a phenylethyl fragment gave compound **11** and resulted in good antiproliferative activity against HePG2, MCF-7, and PC3 cell lines, with IC_50_ values of (34.32, 19.69, and 30.90 µM) and (20.43, 14.55 and 18.75 µM), respectively. Introduction of a 4-methoxyl moiety in compound **11** afforded derivative **12**, which displayed weak antiproliferative activity. The cytotoxic activity of the new compounds was also examined against normal W138 fibroblast cell to study the safety of the newly synthesised compounds, using (MTT) colorimetric assay (Table 1). The tested compounds did not display cytotoxicity towards W138 cells (IC_50_ values of 59.49–204.00 µM) compared to doxorubicin (IC_50_ values of 55.29 µM).

#### 2.2.2. EGFR TK inhibition assay

The most active derivatives, that is, **2**, **3**, **8**, **10**, and **11**, were subjected to the EGFR TK inhibition assay^7^^,^^10^^,^^23–25^. The results revealed that several of the tested compounds were promising EGFR TK inhibitors (Table 2). It was evident that compounds **2**, **3**, **10**, and **11** exhibited strong inhibitory activities against EGFR (IC_50_ values of 1.09, 0.93, 1.12, and 0.81, respectively). Notably, this activity was comparable to that of the reference drug gefitinib (IC_50_ value of 0.90 µM). It was observed that all of the tested derivatives showed 50% inhibition against EGFR of less than 1.2 µM, except compound **8**, which was found to be the least effective EGFR inhibitor (IC_50_ = 4.24 µM). It was determined that compounds incorporating a phenethyl moiety, such as derivative **11**, displayed higher inhibitory activity against EGFR than the corresponding compounds containing a benzyl fragment (e.g. **10**) or a propanol group (e.g. **8**).

**Table 2.** IC_50_ values of compounds **2**, **3**, **8**, **10**, and **11** against EGFR.

	2	3	8	10	11	Gefitinib
EGFR kinase inhibition IC_50_ (µM)	1.09	0.93	4.24	1.12	0.81	0.90

#### 2.2.3. Caspase-3 assay and induction of apoptosis

**Figure 4.**
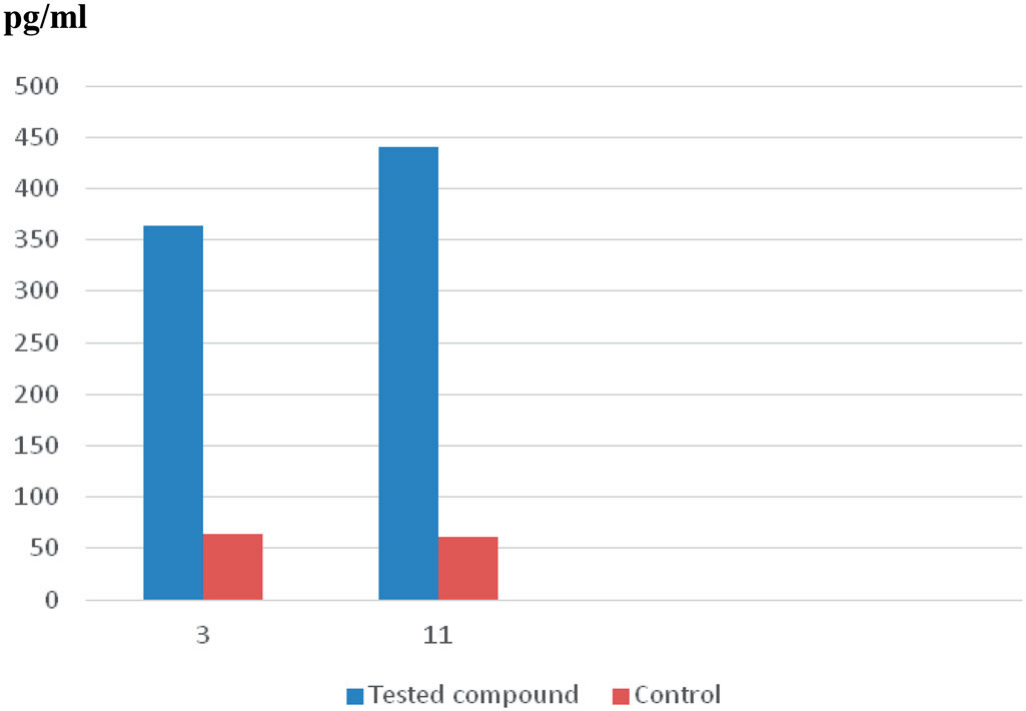
Caspase-3 enzyme assay for compounds **3** and **11**.

Caspases are critical mediators of programmed cell death, that is, apoptosis^49^. Caspase-3 is important in processes involving dissociation of the cell and the formation of the apoptotic element; therefore, it is regarded as one of the best biochemical hallmarks of apoptosis^49^. This, to examine the apoptotic activity of compounds **3** and **11**, the level of caspase-3 was measured after treating the HCT-116 and MCF-7 cells with **3** and **11**, respectively (Table 3). The concentration of active caspase-3 was measured using the ELISA technique^49^. In addition, the fluorescence density produced by the tested compounds is illustrated in Figure 4. Interestingly, compound **3** significantly induced apoptosis in HCT-116 cells after 24 h of treatment. The level of caspase-3 increased 5.7-fold compared to the control. Moreover, a considerable 7.3-fold increase in the caspase-3 level was detected following the treatment of the MCF-7 cells with compound **11**. The bioluminescent intensities of caspase-3 indicated the apoptotic activity of compounds **3** and **11**.

**Table 3.** Effects of compounds **3** and **11** on the levels of human caspase-3.

Compound	Caspase-3 Conc. Pg/ml	Fold
**3**	363.4	5.7
Control/ HCT-116 cell	63.51	
**11**	440.08	7.3
Control/ MCF-7 cell	60.7	

#### 2.2.4. Cell cycle arrest analysis and detection of apoptosis

**Figure 5.**
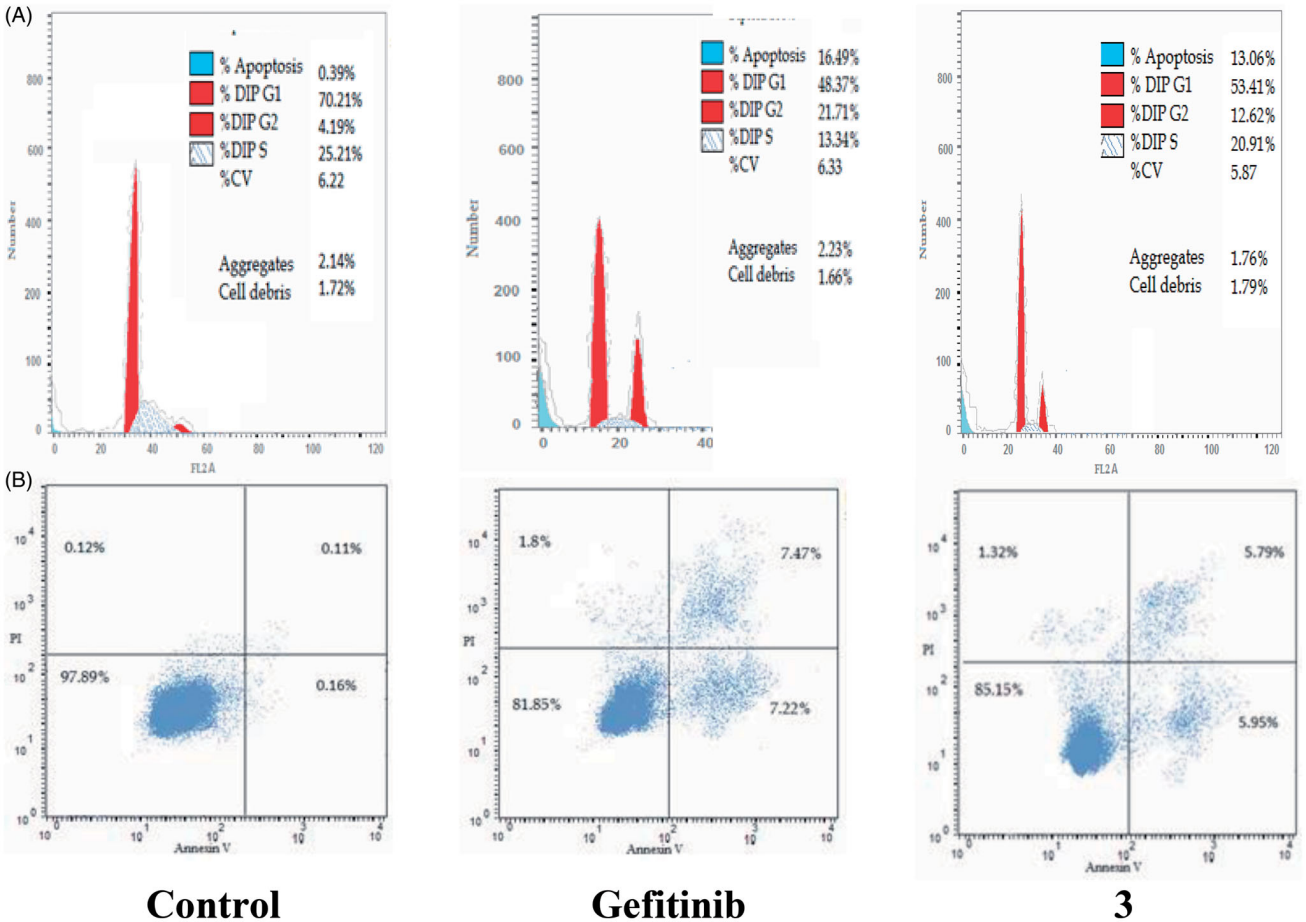
Determination of apoptosis in the HCT-116 cell line and analysis of the cell cycle arrest using flow cytometry. (A) Effect of compound **3** on the cell cycle distribution of HCT-116. (B) Apoptosis effect on the human HCT-116 cell line induced by compound **3**.

**Figure 6.**
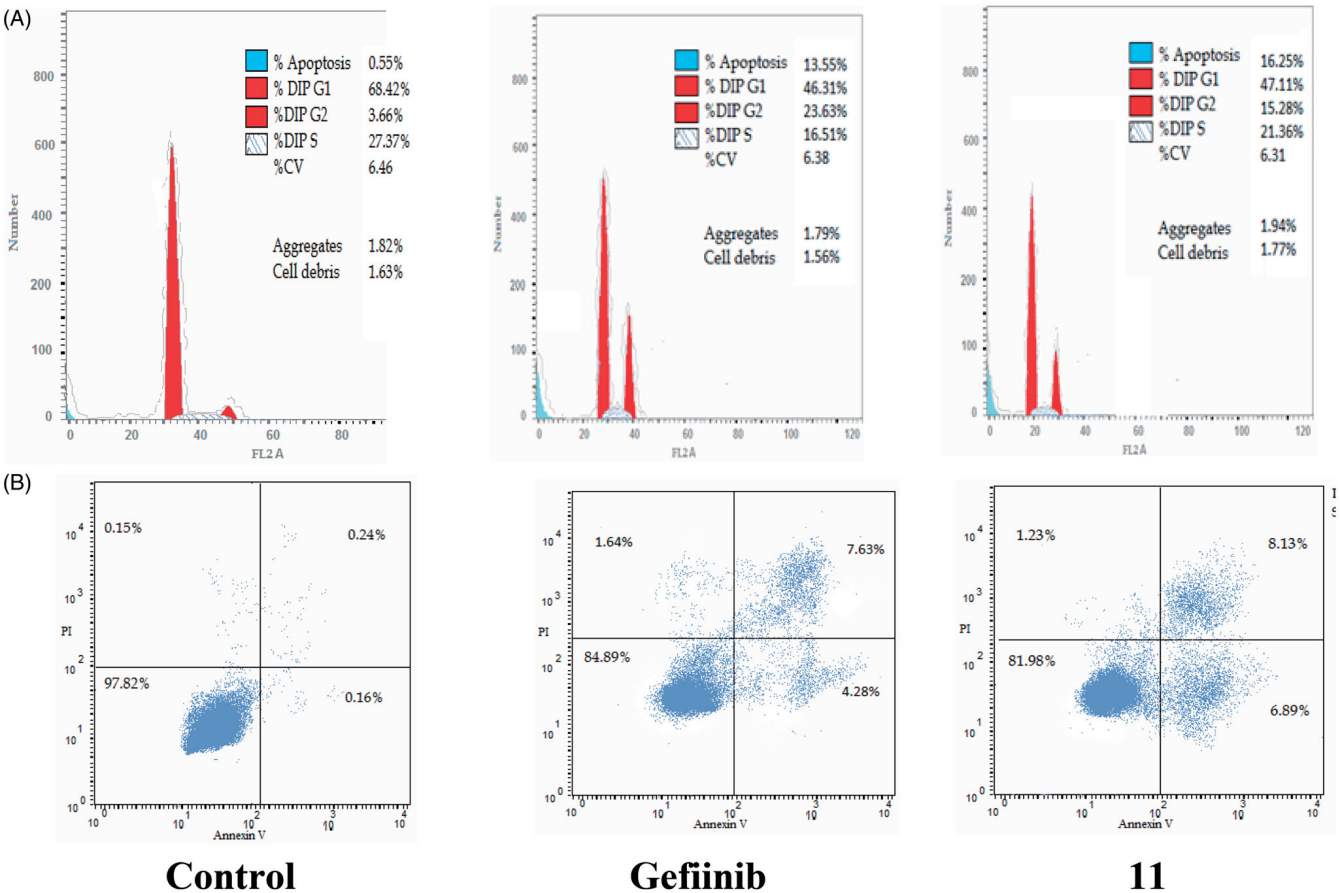
Determination of apoptosis in the MCF-7 cell line and analysis of the cell cycle arrest using flow cytometry. (A) Effect of compound **11** on the cell cycle distribution of MCF-7. (B) Apoptosis effect on the human MCF-7 cell line induced by compound **11**.

**Figure 7.**
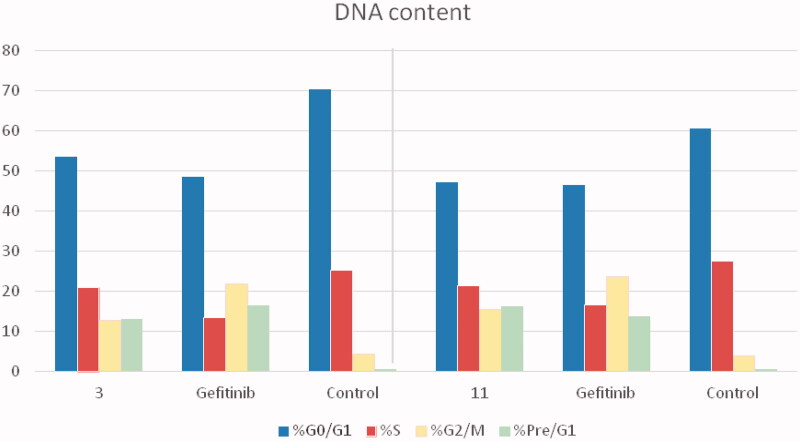
Cell cycle arrest analysis of compounds **3** and **11** in comparison with gefitinib.

**Figure 8.**
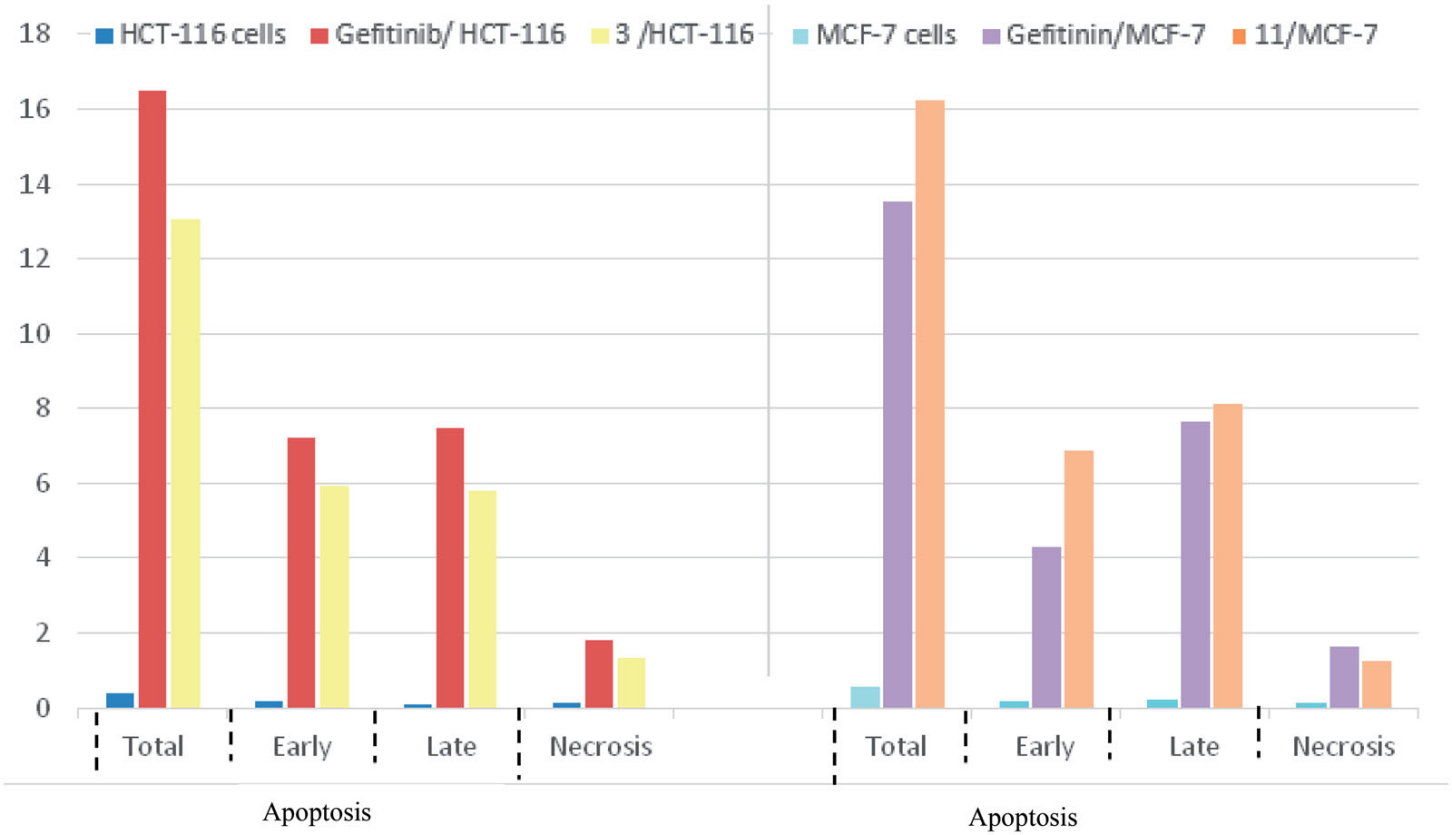
Percentage of apoptosis for compounds **3** and **11** in comparison with gefitinib and control cells.

The cell cycle is a sequence of growth and development steps that lead to DNA replication and cell division. It consists of four distinct phases: the G1 phase, S phase (synthesis), G2 phase, and M phase^23^^,^^24^^,^^50–52^. Apoptosis, that is, programmed cell death, is considered an important target of the most anti-tumour agents, resulting in G2/M arrest^23^^,^^24^^,^^50–52^. Our promising derivatives **3** and **11** were subjected to cell cycle analysis and an apoptotic assay to investigate their roles in the cell cycle progression of HCT-116 and MCF-7 cells, respectively. To better characterise the mode of cell death induced by the tested compounds, following treatment of the HCT-116 and MCF-7 cells with compounds **3** and **11** at a concentration of 10 µM for 24 h, respectively, the cells were stained with propidium iodide (PI). The DNA contents were measured by flow cytometry (Tables 4 and 5; Figures 5–8). Compared with the control, which was treated with DMSO, following treatment of HCT-116 and MCF-7 cells with compounds **3** and **11**, the cell proportion at the S phase decreased to 20.91% and 21.36%, respectively. In addition, compounds **3** and **11** increased the cell proportion at the G2/M phase to 12.62% and 15.28%, respectively, compared to the control cells (4.19% and 3.66%, correspondingly). These results indicated that the cells were arrested at the G2/M phase. Furthermore, the pre-G1 population was detected following treatment with compounds **3** and **11** (13.06% and 16.25% compared to 0.39% and 0.55% in the control cells, respectively). Moreover, annexin-5/PI staining^23^^,^^24^^,^^52^ was performed for compounds 3 and 11 using HCT-116 and MCF-7 cells. A comparison was made to the control (DMSO) and reference (gefitinib). The gefitinib results showed an early apoptosis of 7.22% (HCT-116) and 4.28% (MCF-7), whereas the values for late apoptosis were 7.47% (HCT-116) and 7.63% (MCF-7). The results demonstrated in Table 5, and Figure 8 suggest an increase in the early apoptosis from 0.16% (control sample in DMSO) to 5.95% for compound **3**. In contrast, derivative **11** showed an increase in the early apoptosis to 6.89%. Compounds **3** and **11** increased the late apoptosis from 0.11% (DMSO) to 5.79% and 8.13%, respectively. It was also evident that **3** and **11** preferentially activated the apoptotic pathway rather than the necrotic pathway. This induced action was the result of the cell cycle arrest at the G2/M phase.

**Table 4.** Analysis of the effects of compounds **3** and **11** on the cell cycle progression in HCT-116 and MCF-7 cells, respectively, using flow cytometry.

	%G0/G1		%S	%G2/M	%Pre/G1
Cell cycle arrest analysis in HCT-116 (µM)					
Control	70.21		25.21	4.19	0.39
Gefitinib	48.37		13.34	21.71	16.49
**3**	53.41		20.91	12.62	13.06
Cell cycle arrest analysis in MCF-7 (µM)					
Control	66.42	27.37		3.66	0.55
Gefitinib	46.31	16.51		23.63	13.55
**11**	47.11	21.36		15.28	16.25

**Table 5.** Apoptotic activity of compounds **3** and **11** in HCT-116 and MCF-7 cell lines, respectively.

Compound	Total	Early apoptosis	Late apoptosis	Necrosis
Control	0.39	0.16	0.11	0.12
Gefitinib	16.49	7.22	7.47	1.8
**3**	13.06	5.95	5.79	1.32
Control	0.55	0.16	0.24	0.15
Gefitinib	13.55	4.28	7.63	1.64
**11**	16.25	6.89	8.13	1.23

#### 2.2.5. Molecular modelling study

**Figure 9.**
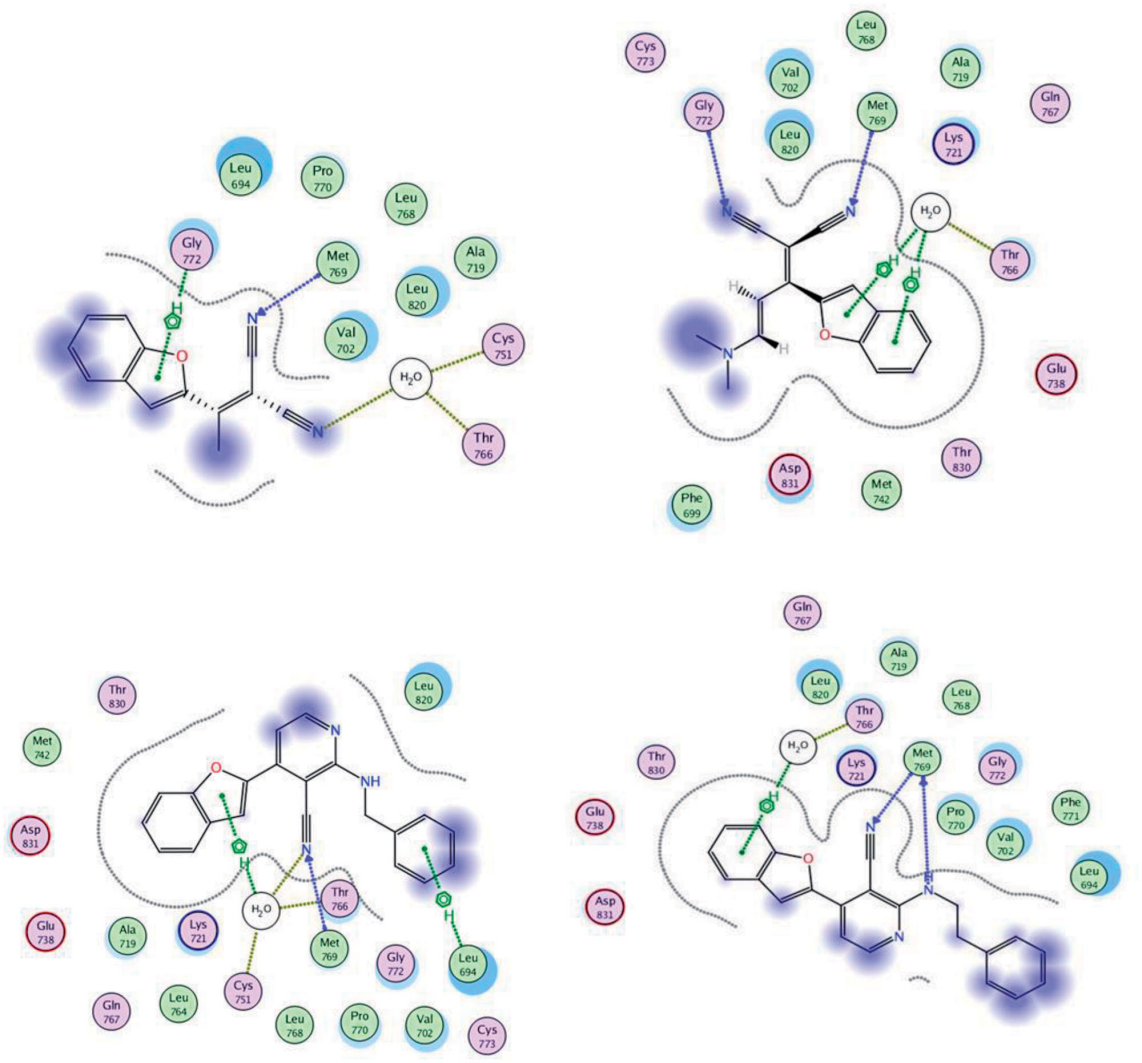
2D binding modes and residues involved in the recognition of the most active compounds docked and minimised in the EGFR binding pocket: Compound **2** (upper left panel), **3** (upper right panel), **10** (lower left panel), and **11** (lower right panel).

Molecular modelling is a tool used to inspect bioactive molecules within a putative binding site of a particular enzyme or receptor^53–57^. It can also be employed for studying the molecular structure and structural activity relationship of different molecules^6^^,^^58^^,^^59^. In this study, the MOE 2008.10 software obtained from the Chemical Computing Group Inc. (Montreal, QC, Canada) was used for the docking protocol. The docked compounds and the co-bound inhibitor were docked into the putative binding site of the protein to generate an appropriate binding orientation. Molecular docking of the most active compounds **2**, **3**, **10**, and **11** was conducted to explore their binding modes and interactions with the constitutive amino acids in the active site of EGFR. Molecular operating environment (MOE) software version 2008.10 was used for the analysis (Figure 9). The crystal structure of the EGFR TK receptor in complex with erlotinib was obtained from the RCSB protein data bank (PDB ID: 1M17) and was utilised to establish the starting docking model of EGFR TK^60^. The quinazoline core of the erlotinib inhibitor exhibited a hydrogen bond with Met769^60^. Erlotinib was subjected to one run of docking into the binding site to verify and validate the docking process. Docking of **2**, **3**, **10**, and **11** revealed that all compounds fit into the enzyme active site almost at the same position as erlotinib (Figure 9). One of the nitrile groups of compound **2** (*S* = −8.53 kcal/mol) is bound to the active site of EGFR TK through hydrogen bonding with the vital amino acid Met769. The second nitrile moiety interacted with the amino acid residues Thr766 and Cys751 through water-mediated hydrogen bonding.

Moreover, the benzofuran core is connected to Gly772 by cation–H interactions. Derivative **3** (*S* = −9.38 kcal/mol) interacted with residues Met769 and Gly772 via hydrogen bonding using its two nitrile moieties. The benzofuran ring of **3** bound to Thr766 by water-mediated arene–H interactions. The nitrile moiety in compound **10** (*S* = −9.61 kcal/mol) connected to Met769 by hydrogen bonding, while the benzofuran ring interacted with amino acid residues Thr766 and Cys751 via water-mediated cation–π bonding. In addition, the benzyl fragment is bound to residue Leu694 by cation–π interactions. The best docking score was achieved for compound **11** (−10.38 kcal/mol). **11** bound to the active site of EGFR TK through two hydrogen bonds to the critical amino acid Met769 using the N atom of the nitrile moiety and the N atom of phenethylamine. Lastly, the benzofuran core bound to Thr766 by water-mediated arene–H interactions (Figure 9).

## 3. Conclusion

To develop potent anti-tumour agents, a series of cyanobenzofuran hybrids were designed and synthesised in this work. The *in vitro* antiproliferative activity of the prepared compounds was evaluated for HePG2, HCT-116, MCF-7, PC3, and HeLa cancer cell lines. The biological results revealed that compounds **2**, **3**, **10,** and **11** exhibited a broad spectrum antiproliferative activity against selected cell lines. Moreover, the most active derivatives were further evaluated for their inhibitory activity against EGFR kinase. Compounds **3** and **11** displayed significant EGFR TK inhibitory activity (IC_50_ 0.93 and 0.81 µM, respectively) even compared to the reference drug gefitinib (IC_50_ 0.9 µM). Compounds **2** and **10** also showed good EGFR TK inhibitory activity (IC_50_ 1.09 and 1.12 µM, respectively). The apoptosis assay and cell cycle analysis results demonstrated that derivatives **3** and **11** induced apoptosis of cancer cells and arrested the cell cycle at the G2/M phase. In addition, **3** and **11** led to an increase in caspase-3 by 5.7- and 7.3-fold, respectively. These outcomes indicated that the potent pro-apoptotic activity of compounds **3** and **11** was a result of the induction of the intrinsic apoptotic pathway rather than the necrotic pathway. Compared to erlotinib, the molecular docking analysis of the most active compounds **2**, **3**, **10**, and **11** showed good fitting and suitable interactions with the key amino residues in the binding site of the EGFR kinase. The presence of the cyano group in the compounds enabled hydrogen bonding interactions with the Met769 amino acid. Additionally, the benzofuran moiety exhibited van der Waals interactions with the EGFR binding site. Based on these findings, it can be concluded that derivatives **3** and **11** are promising scaffolds for further modification and optimisation to obtain potent and selective anti-tumour agents with EGFR inhibitory activity.

## 4. Experimental

### 4.1. Chemistry

All melting points (°C) were recorded using a Fisher–John melting point apparatus and were uncorrected. The IR spectra were determined for KBr discs on a Thermo Fischer Scientific Nicolet IS10 spectrometer (wavenumber in cm^−1^) at the Faculty of Pharmacy, Mansoura University, Egypt. The ^1^H NMR spectra were obtained in DMSO-d_6_ or CDCl_3_ employing a Jeol 500 MHz spectrometer at the Faculty of Science, Mansoura University, Egypt. The ^13^C NMR spectra were obtained in DMSO-d_6_ using a Jeol 500 MHz spectrometer at the Faculty of Science, Mansoura University, Egypt. Electron ionisation mass spectrometry (EI MS) was performed on a Hewlett Packard 5988 spectrometer at the Al-Azhar University, Cairo, Egypt. Microanalyses (C, H, N) were conducted at the Microanalytical Unit, Cairo University, and the results were within ±0.4% of the theoretical values. The antiproliferative screening of all newly synthesised compounds, enzyme activity inhibition assay, caspase-3 assay, apoptosis induction analysis, and cell cycle analysis was conducted at the Holding Company for Biological Products and Vaccines (VACSERA), Cairo, Egypt.

#### 4.1.1. 2-(1-Benzofuran-2-yl)ethylidene)malononitrile (2)

A solution of 2-acetylbenzofuran (**1**) (0.32 g, 2.0 mmol) and CH_2_(CN)_2_ (malononitrile) (0.132 g, 2.0 mmol) in EtOH (15 ml) was refluxed for 24 h. Yellow crystals were formed after cooling and evaporating ethanol under reduced pressure. The crystals were collected by filtration and recrystallized from EtOH to afford the titled compound crystalline yellow needles.

Yield = 90%, m.p. = 140–142 °C, crystalline yellow needles; IR (KBr, cm^−1^): 3018 (=C–H), 2222 (CN), 1610 (C=C); ^1^H NMR (500 MHz, DMSO-d_6_): *δ* = 2.63 (s, 3H, CH_3_), 7.42 (dd, *J* = 8.9, 8.9 Hz, 1H, ArH), 7.60 (dd, *J* = 9.3, 9.3 Hz, 1H, ArH), 7.70 (d, *J* = 10.5 Hz, 1H, ArH), 7.87 (d, *J* = 9.6 Hz, 1H, ArH), 8.08 (s, 1H, ArH); ^13^C NMR (125 MHz, DMSO-d_6_): *δ* = 19.38, 78.23, 111.56, 113.24, 113.60, 117.39, 123.23, 124.28, 126.83, 129.83, 149.91, 155.11, 156.94; MS (EI) *m/z* (C_13_H_8_N_2_O): 209.08 (M + 1, 60.5%), 208 (M^+^, 39.53%), 18.05 (20.28%), 167.08 (13.06%), 153.08 (7.61%), 143.04 (4.71%), 118.06 (6.95%), 94.07 (15.20%), 67.08 (6.83%), 44.03 (25.09%); Elemental Analysis for C_13_H_8_N_2_O, Calcd.: C, 74.8; H, 3.87; N, 13.45; Found: C, 74.98; H, 3.88; N, 13.46.

#### 4.1.2. (E)-2-(1-(benzofuran-2-yl)-3-(dimethylamino)allylidene)malononitrile (3)

DMFDMA (2.69 g, 3 ml, 22.57 mmol) was added to compound **2** (0.1 g, 0.48 mmol) and stirred at room temperature overnight in a solvent free environment. The mixture was then washed with diethyl ether and filtered to give crystalline yellow needles. Yield= 60%, m.p. = 161–163 °C, crystalline yellow needles, IR (KBr, cm^−1^): 3060 (=C–H), 2197 (CN), 1610 (C=C); ^1^H NMR (500 MHz, DMSO-d_6_): *δ* = 3.09 (s, 3H, CH_3_), 3.23 (s, 3H, CH_3_), 5.70 (d, *J* = 12.0 Hz, 1H, CH), 7.35 (t, *J* = 7.4 Hz, 1H, ArH), 7.43 (s, 1H, ArH), 7.46 (m, 1H, ArH), 7.68 (d, *J* = 8.7 Hz, 1H, ArH), 7.72 (d, *J* = 12.1 Hz, 1H, CH), 7.78 (d, *J* = 7.7 Hz, 1H, ArH); ^13^C NMR (125 MHz, DMSO-d_6_): *δ* = 37.86, 45.77, 95.72, 111.81, 112.18, 122.28, 123.69, 126.46, 127.42, 148.44, 154.83, 156.05, 157.59; Elemental Analysis for C_16_H_13_N_3_O, Calcd.: C, 72.8; H, 4.98; N, 15.96; Found: C, 72.98; H, 4.8; N, 15.97.

#### 4.1.3. General procedure for the preparation of 4-(benzofuran-2-yl)-2-(substituted amino)nicotinonitriles 4–12

A mixture of compound **3** (0.2 g, 0.7 mmol) and an appropriate primary amine (2 ml) was refluxed for one h and stirred at room temperature overnight. The reaction mixture was diluted with CH_2_Cl_2_ (20 ml) and washed with brine (5 ml). The aqueous layer was extracted with CH_2_Cl_2_ (20 ml × 3), and the organic extracts were dried over anhydrous Na_2_SO_4_, filtered, and evaporated under reduced pressure. The obtained residue was purified by column chromatography (SiO_2_:CH_2_Cl_2_/MeOH = 30:1) to give the titled products **4–12** as yellow solids.

##### 4.1.3.1. 4-(Benzofuran-2-yl)-2-(ethylamino)nicotinonitrile (4)

Yield = 40%, m.p. = 155–158 °C, yellow powder, IR (KBr, cm^−1^): 3350 (NH), 2215 (CN); ^1^H NMR (500 MHz, DMSO-d_6_): *δ* = 1.15 (t, *J* = 7.1 Hz, 3H, CH_3_), 3.46 (q, *J =* 5.6 Hz, 2H, CH_2_), 7.15 (d, *J* = 5.3 Hz, 1H, NH), 7.25 (t, *J* = 5.4 Hz, 1H, ArH), 7.35 (t, *J* = 7.8 Hz, 1H, ArH), 7.47 (dd, *J* = 11.4, 11.4 Hz, 1H, ArH), 7.69 (d, *J* = 8.2 Hz, 1H, ArH), 7.83 (d, *J* = 7.8 Hz, 1H, ArH), 7.86 (s, 1H, ArH), 8.36 (d, *J* = 5.3 Hz, 1H, ArH); Elemental Analysis for C_16_H_13_N_3_O, Calcd.: C, 72.8; H, 4.98; N, 15.96; Found: C, 72.97; H, 4.8; N, 15.95.

##### 4.1.3.2. 4-(Benzofuran-2-yl)-2-(propylamino)nicotinonitrile (5)

Yield = 88%, m.p. = 118–120 °C, yellow powder, IR (KBr, cm^−1^): 3361 (NH), 2212 (CN), 1583 (C=C); ^1^H NMR (500 MHz, DMSO-d_6_): *δ* = 0.90 (t, *J* = 7.4 Hz, 3H, CH_3_), 1.59 (sextet, *J* = 7.4 Hz, 2H, CH_2_), 3.39 (td, *J* = 7.1, 7.5 Hz, 2H, CH_2_), 7.15 (d, *J* = 5.2 Hz, 1H, NH), 7.26 (t, *J* = 5.6 Hz, 1H, ArH), 7.36 (t, *J* = 7.4 Hz, 1H, ArH), 7.47 (m, 1H, ArH), 7.70 (d, *J* = 8.7 Hz, 1H, ArH), 7.83 (d, *J* = 7.7 Hz, 1H, ArH), 7.87 (s, 1H, ArH), 8.37 (d, *J* = 5.2 Hz, 1H, ArH); Elemental Analysis for C_17_H_15_N_3_O, Calcd.: C, 73.63; H, 5.45; N, 15.15; Found: C, 73.64; H, 5.46; N, 15.17.

##### 4.1.3.3. 4-(Benzofuran-2-yl)-2-(butylamino)nicotinonitrile (6)

Yield = 60%, m.p. = 112–114 °C, yellow powder, IR (KBr, cm^−1^): 3354 (NH), 2865 (C–H), 2215 (CN), 1588 (C=C); ^1^H NMR (500 MHz, DMSO-d_6_): *δ* = 0.90 (t, *J* = 7.4 Hz, 3H, CH_3_), 1.32 (sextet, *J* = 7.5 Hz, 1H, ArH), 1.55 (tt, *J* = 7.5 Hz, 2H, CH_2_), 3.42 (d, *J* = 6.3 Hz, 2H, CH_2_), 7.13 (d, *J* = 5.3 Hz, 1H, NH), 7.22 (s, 1H, ArH), 7.35 (d, *J* = 7.8 Hz, 1H, ArH, 7.54 (m, 1H, ArH), 7.69 (d, *J* = 8.8 Hz, 1H, ArH), 7.82 (d, *J* = 7.8 Hz, 1H, ArH), 7.85 (d, *J* = 0.7 Hz, 1H, ArH), 8.35 (d, *J* = 5.2 Hz, 1H, ArH); ^13^C NMR (125 MHz, DMSO-d_6_): *δ* = 13.49, 19.29, 30.63, 40.33, 83.8, 107.77, 108.59, 111.18, 116.42, 122.15, 123.52, 126.49, 127.43, 140.12, 149.75, 152.57, 153.91, 159.23; Elemental Analysis for C_18_H_17_N_3_O, Calcd.: C, 74.20; H, 5.88; N, 14.42; Found: C, 74.21; H, 5.89; N, 14.40.

##### 4.1.3.4. 4-(Benzofuran-2-yl)-2-(2-hydroxyethylamino)nicotinonitrile (7)

Yield= 70%, m.p. = 164–166 °C, yellow powder, IR (KBr, cm^−1^): 3000 (OH), 3344 (NH), 2938, 2219 (CN), 1591 (C=C); ^1^H NMR (500 MHz, DMSO-d_6_): *δ* = 3.51 (td, *J* = 7.5, 5.1 Hz, 2H, CH_2_), 3.56 (td, *J* = 7.5, 5.2 Hz, 2H, CH_2_), 4.79 (t, *J* = 5.2 Hz, 1H, OH), 7.04 (t, *J* = 5.1 Hz, 1H, NH), 7.17 (d, *J* = 5.3 Hz, 1H, ArH), 7.35 (t, *J* = 7.4 Hz, 1H, ArH), 7.47 (m, 1H, ArH), 7.70 (d, *J* = 8.8 Hz, 1H, ArH), 7.83 (d, *J* = 7.7 Hz, 1H, ArH), 7.87 (d, *J* = 0.8 Hz, 1H, ArH), 8.36 (d, *J* = 5.2 Hz, 1H, ArH); ^13^C NMR (125 MHz, DMSO-d_6_): *δ* = 43.52, 59.28, 84.58, 108.42, 109.02, 111.55, 116.72, 122.53, 123.98, 126.89, 127.76, 140.43, 150.02, 152.84, 154.28, 159.59; Elemental Analysis for C_16_H_13_N_3_O_2_, Calcd.: C, 68.81; H, 4.69; N, 15.05; Found: C, 68.82; H, 4.67; N, 15.04.

##### 4.1.3.5. 4-(Benzofuran-2-yl)-2-(3-hydroxypropylamino)nicotinonitrile (8)

Yield = 88%, m.p. = 124–126 °C, yellow powder, IR (KBr, cm^−1^): 3100 (OH), 3343 (NH), 2218 (CN), 1594 (C=C); ^1^H NMR (500 MHz, DMSO-d_6_): *δ* = 1.73 (p, *J* = 6.5 Hz, 2H, CH_2_), 3.50 (m, 4H, 2CH_2_), 5.75 (s, 1H, OH), 7.15 (d, *J* = 5.3 Hz, *J* 1H, NH), 7.24 (t, *J* = 5.4 Hz, 1H, ArH), 7.34 (dd, *J* = 11.4, 11.4 Hz, 1H, ArH), 7.46 (m, 1H, ArH), 7.69 (d, *J* = 7.9 Hz, 1H, ArH), 7.82 (d, = 7.7 Hz, 1H, ArH), 7.86 (s, 1H, ArH), 8.36 (d, *J* = 5.3 Hz, 1H, ArH); Elemental Analysis for C_17_H_15_N_3_O_2_, Calcd.: C, 69.61; H, 5.15; N, 14.33; Found: C, 69.63; H, 5.14; N, 14.32.

##### 4.1.3.6. 4-(Benzofuran-2-yl)-2-(cyclohexylamino)nicotinonitrile (9)

Yield = 50%, m.p. = 186–188 °C, yellow powder, IR (KBr, cm^−1^): 3355 (NH), 2210 (CN), 1579 (C=C); ^1^H NMR (500 MHz, DMSO-d_6_): *δ* = 1.14 (m, 1H, CH), 1.31 (m, 2H, CH_2_), 1.42 (m, 2H, CH_2_), 1.61 (d, *J* = 12.7 Hz, 1H, CH), 1.73 (d, *J* = 13.1 Hz, 2H, CH_2_), 1.87 (d, *J* = 9.8 Hz, 2H, CH_2_), 4.00 (m, 1H, CH), 6.66 (d, *J* = 7.9 Hz, 1H, NH), 7.15 (d, *J* = 5.2 Hz, 1H, ArH), 7.35 (t, *J* = 7.4 Hz, 1H, ArH), 7.46 (dd, *J* = 11.7, 4.3 Hz, 1H, ArH), 7.69 (d, *J* = 8.2 Hz, 7.83 (d, *J* = 7.7 Hz, 1H, ArH), 7.86 (s, 1H, ArH), 8.36 (d, *J* = 5.3 Hz, 1H, ArH); ^13^C NMR (125 MHz, DMSO-d_6_): *δ* = 24.8, 25.26, 31.93, 49.79, 84.42, 108.33, 108.98, 111.52, 117.61, 122.50, 123.86, 126.84, 127.76, 140.64, 150.04, 152.87, 154.25, 158.75; Elemental Analysis for C_20_H_19_N_3_O, Calcd.: C, 75.69; H, 6.03; N, 13.24; Found: C, 75.68; H, 6.01; N, 13.25.

##### 4.1.3.7. 4-(Benzofuran-2-yl)-2-(benzylamino)nicotinonitrile (10)

Yield = 43%, m.p. = 180–182 °C, yellow powder. IR (KBr, cm^−1^): 3029 (=C–H), 3369 (NH), 2211 (CN), 1580 (C=C); ^1^H NMR (500 MHz, DMSO-d_6_): *δ* = 4.56 (d, *J* = 6.0 Hz, 2H, CH_2_), 7.17 (d, *J* = 5.2 Hz, 1H, NH), 7.21 (dd, *J* = 10.0, 10.0 Hz, 1H, ArH), 7.30 (s, 1H, ArH), 7.31 (d, *J* = 7.4 Hz, 1H, ArH), 7.33 (s, 2H. ArH), 7.35 (d, *J* = 2.6 Hz, 1H, ArH), 7.47 (s, 1H, ArH), 7.69 (d, *J* = 7.8 Hz, 1H, ArH), 7.83 (d, *J* = 7.7 Hz, 1H, ArH), 7.87 (d, *J* = 6.0 Hz, 1H, ArH), 7.88 (s, 1H, ArH), 8.32 (d, *J* = 5.2 Hz, 1H, ArH); ^13^C NMR (125 MHz, DMSO-d_6_): *δ* = 44.03, 84.62, 108.69, 109.06, 111.53, 116.65, 122.52, 123.88, 126.65, 126.87, 127.08, 127.75, 128.18, 139.94, 140.53, 150.01, 152.83, 154.29, 159.41; Elemental Analysis for C_21_H_15_N_3_O, Calcd.: C, 77.52; H, 4.65; N, 12.91; Found: C, 77.53; H, 4.64; N, 12.93.

##### 4.1.3.8. 4-(Benzofuran-2-yl)-2-(phenethylamino)nicotinonitrile (11)

Yield = 56%, m.p. = 157–159 °C, yellow powder, IR (KBr, cm^−1^): 3022 (=C–H), 3370 (NH), 2209 (CN), 1588 (C=C); ^1^H NMR (500 MHz, DMSO-d_6_): *δ* = 2.90 (t, *J* = 6.2 Hz, 2H, CH_2_Ph), 3.66 (td, *J* = 6.2, 5.3 Hz, 2H, CH_2_N), 7.18 (d, *J* = 5.3 Hz, NH), 7.21 (t, *J* = 7.2 Hz, 1H, ArH), 7.25 (s, 1H, ArH), 7.26 (s, 1H, ArH), 7.30 (s, 1H, ArH), 7.31 (d, *J* = 3.1 Hz, 1H, ArH), 7.33 (s, 1H, ArH), 7.36 (t, *J* = 7.6 Hz, 1H, ArH), 7.47 (m, 1H, ArH), 7.70 (d, *J* = 8.4 Hz, 1H, ArH), 7.83 (d, *J* = 7.8 Hz, 1H, ArH), 7.88 (s, 1H, ArH), 8.40 (d, *J* = 5.2 Hz, 1H, ArH); Elemental Analysis for C_22_H_17_N_3_O, Calcd.: C, 77.86; H, 5.05; N, 12.38; Found: C, 77.87; H, 5.03; N, 12.36.

##### 4.1.3.9. 4-(Benzofuran-2-yl)-2-(4-methoxyphenethylamino)nicotinonitrile (12)

Yield = 66%, m.p. = 156–158 °C, yellow powder, IR (KBr, cm^−1^): 3013 (=C–H), 3352 (NH), 2217 (CN), 158 (C=C); ^1^H-NMR (500 MHz, DMSO-d_6_): *δ* = 2.82 (m, 2H, CH_2_), 3.60 (m, 2H, CH_2_), 3.71 (s, 3H, CH_3_), 6,85 (s, 1H, NH), 6.87 (s, 1H, ArH), 7.15 (s, 1H, ArH), 7.16 (d, *J* = 2.8 Hz, 1H, ArH), 7.17 (s, 1H, ArH), 7.25 (s, 1H, ArH), 7.35(s, 1H, ArH), 7.46 (s, 1H, ArH), 7.69 (d, *J* = 8.4 Hz, 1H, ArH), 7.82 (d, *J* = 7.9 Hz, 1H, ArH), 7.86 (s, 1H, ArH), 8.39 (d, *J* = 5.3 Hz, 1H, ArH); ^13^C NMR (125 MHz, DMSO-d_6_): *δ* = 34.0, 42.83, 54.98, 84.50, 108.34, 108.8, 111.54, 113.79, 116.68, 122.51, 123.89, 126.87, 129.63, 131.40, 140.45, 150.05, 152.97, 154.27, 157.66, 159.46; Elemental Analysis for C_23_H_19_N_3_O_2_, Calcd.: C, 74.78; H, 5.18; N, 11.37; Found: C, 74.77; H, 5.17; N, 11.36.

### 4.2. Biological evaluation

#### 4.2.1. Antiproliferative screening

The *in vitro* antiproliferative activity of all synthesised compounds was evaluated by an MTT assay according to the reported method^47^^,^^48^.

#### 4.2.2. Epidermal growth factor inhibition assay

EGFR enzyme assay was conducted as described in our previous reports^23–25^.

#### 4.2.3. Caspase-3 assay

Sandwich enzyme-linked immunosorbent assay (ELISA) was used to determine the level of active human caspase-3 as previously reported and according to the manufacturer’s instructions^49^.

#### 4.2.4. Cell cycle analysis and induction of apoptosis

##### 4.2.4.1. Flow cytometry analysis of the cell cycle distribution

Cell cycle analysis was performed according to our previous report using the HCT-116 and MCF-7 cell lines stained with the DNA fluorochrome PI and analysed by FACSCalibur flow cytometer^50–52^.

##### 4.4.2.2. Analysis of cellular apoptosis

Apoptosis induction was performed using the HCT-116 and MCF-7 cell lines and well-established Annexin 5-FITC/PI detection kit similar to the report procedure^50–52^. The cell line samples were analysed using FACSCalibur flow cytometer.

### 4.3. Docking study

The molecular modelling calculations and docking studies were performed using the MOE^61^ software version 2008.10 (Chemical Computing Group Inc., Montreal, Quebec, Canada). The X-ray crystallographic structure of EGFR with erlotinib was obtained from the RCSB protein data bank (PDB ID: 1m17).

## Supplementary Material

Supplemental Material
