# Supplementary material for: Design, synthesis, and analysis of antiproliferative and apoptosis-inducing activities of nitrile derivatives containing a benzofuran scaffold: EGFR inhibition assay and molecular modelling study
Source: J Enzyme Inhib Med Chem. 2021 Jul 6;36(1):1488–99. doi: 10.1080/14756366.2021.1946044 (PMC8266232; doi:10.1080/14756366.2021.1946044)
Supplement: Supplemental Material [file IENZ_A_1946044_SM6538.pdf]

Design, synthesis, and analysis of antiproliferative and apoptosis-inducing activities of nitrile derivatives containing a benzofuran scaffold: EGFR inhibition assay and molecular modeling study

Salma Fares[1,2], Khalid B. Selim[1], Fatma E. Goda[1], Magda A. A. El-Sayed[1,3], Nawaf A. AlSaif [4], Mohamed M. Hefnawy [4], Alaa A.-M. Abdel-Aziz[4], Adel S. El-Azab[4]*

[1]*Department of Pharmaceutical Organic Chemistry, Faculty of Pharmacy, Mansoura University, Mansoura 35516, Egypt.*

[2]*Department of Pharmaceutical Chemistry, Faculty of Pharmacy, Delta University for Science and Technology, Gamasa City, Egypt.*

[3]*Department of Pharmaceutical Chemistry, Horus University, New Dammeitta, Egypt*

[4]*Department of Pharmaceutical Chemistry, College of Pharmacy, P.O. Box 2457, King Saud University, Riyadh 11451, Saudi Arabia*

1. NMR Data

2. Experimental procedures

1. NMR Data

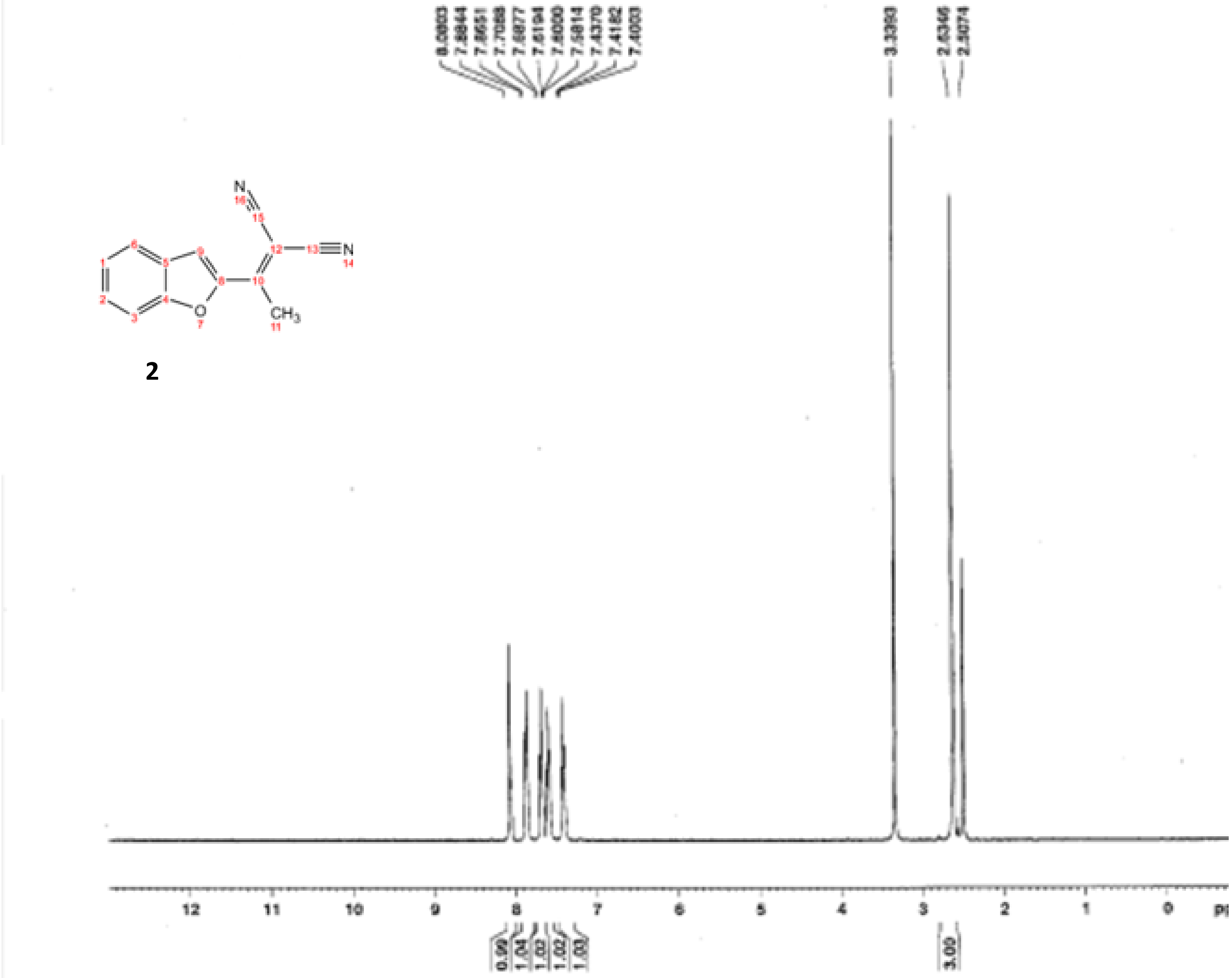

Chart 1. ^{1}H-NMR spectrum (500 MHz, DMSO-d_6) for compound **2**.

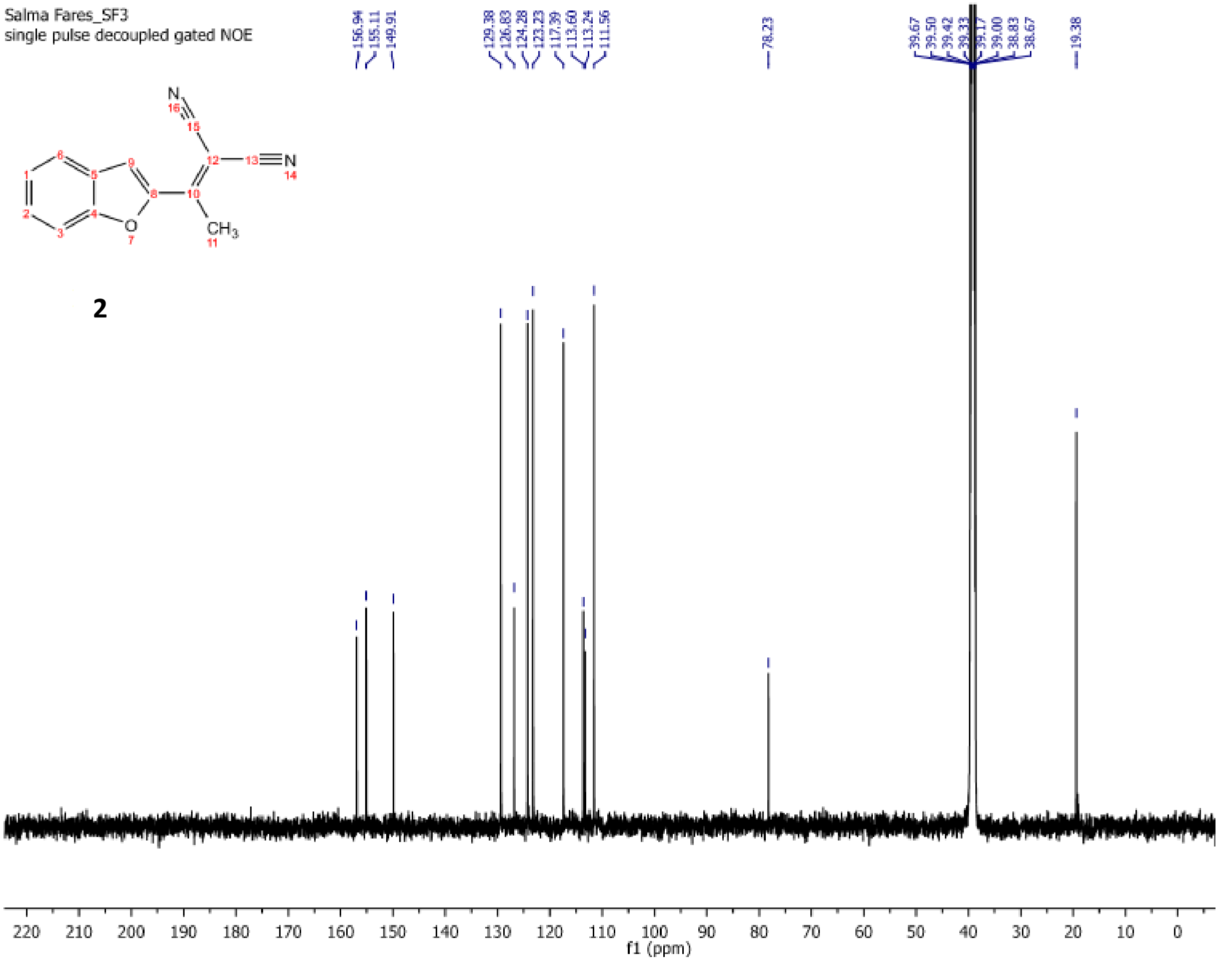

Chart 2. ^{13}C-NMR spectrum (125 MHz, DMSO-d$_6$) for compound **2**.

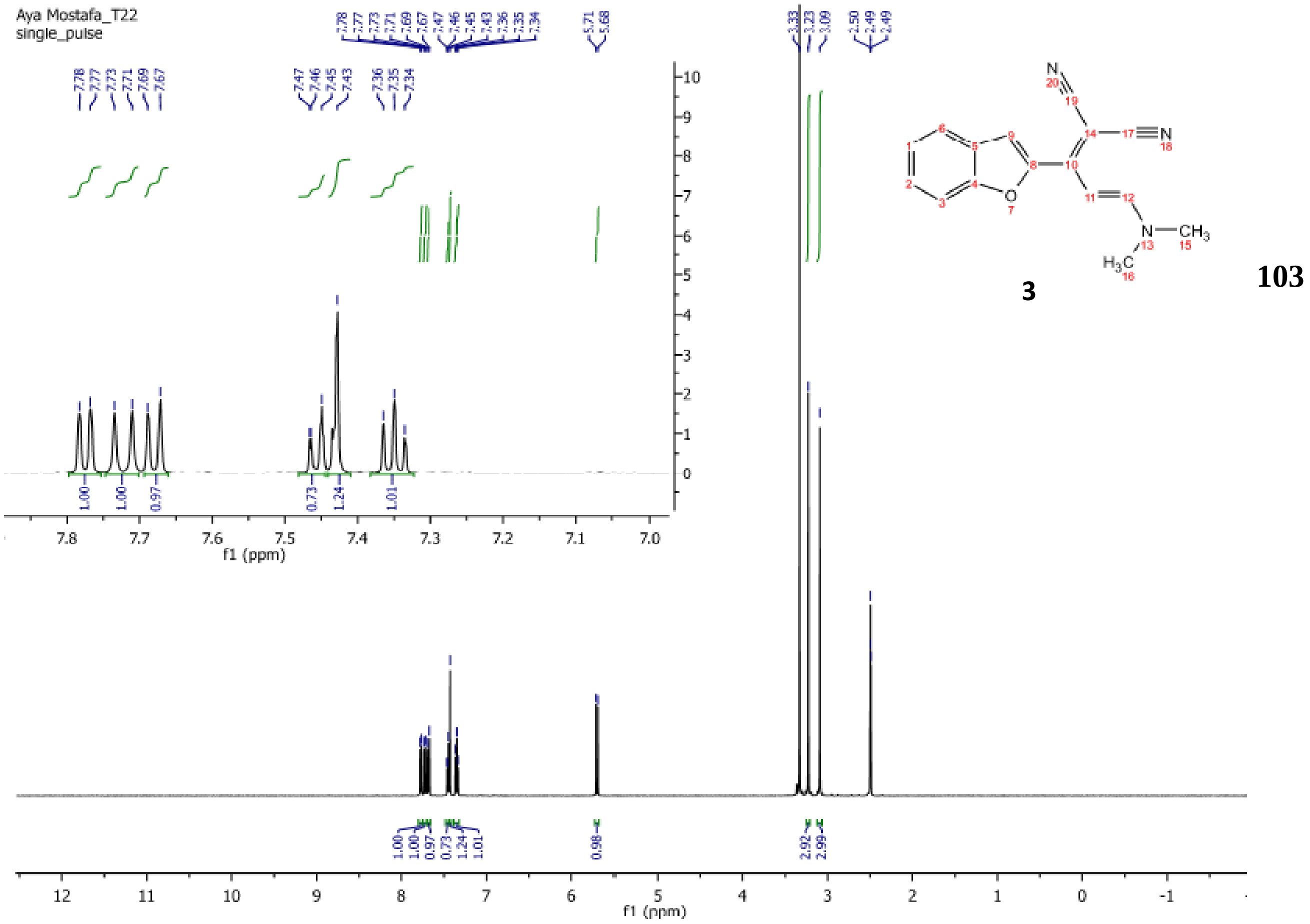

.

Chart 3. ^{1}H-NMR spectrum (500 MHz, DMSO-d_6) for compound **3**.

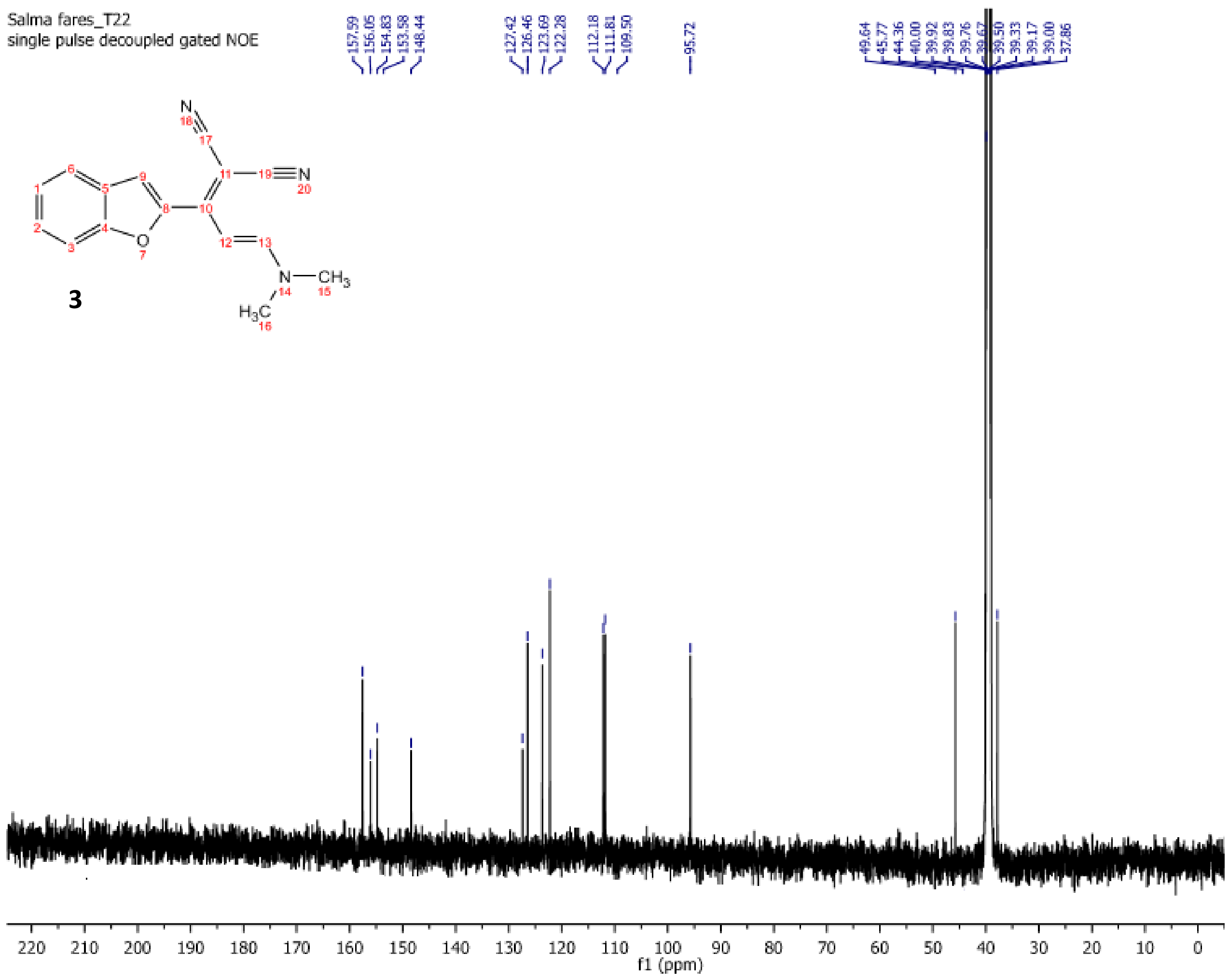

Chart 4. ^{13}C-NMR spectrum (125 MHz, DMSO-d_6) for compound **3**.

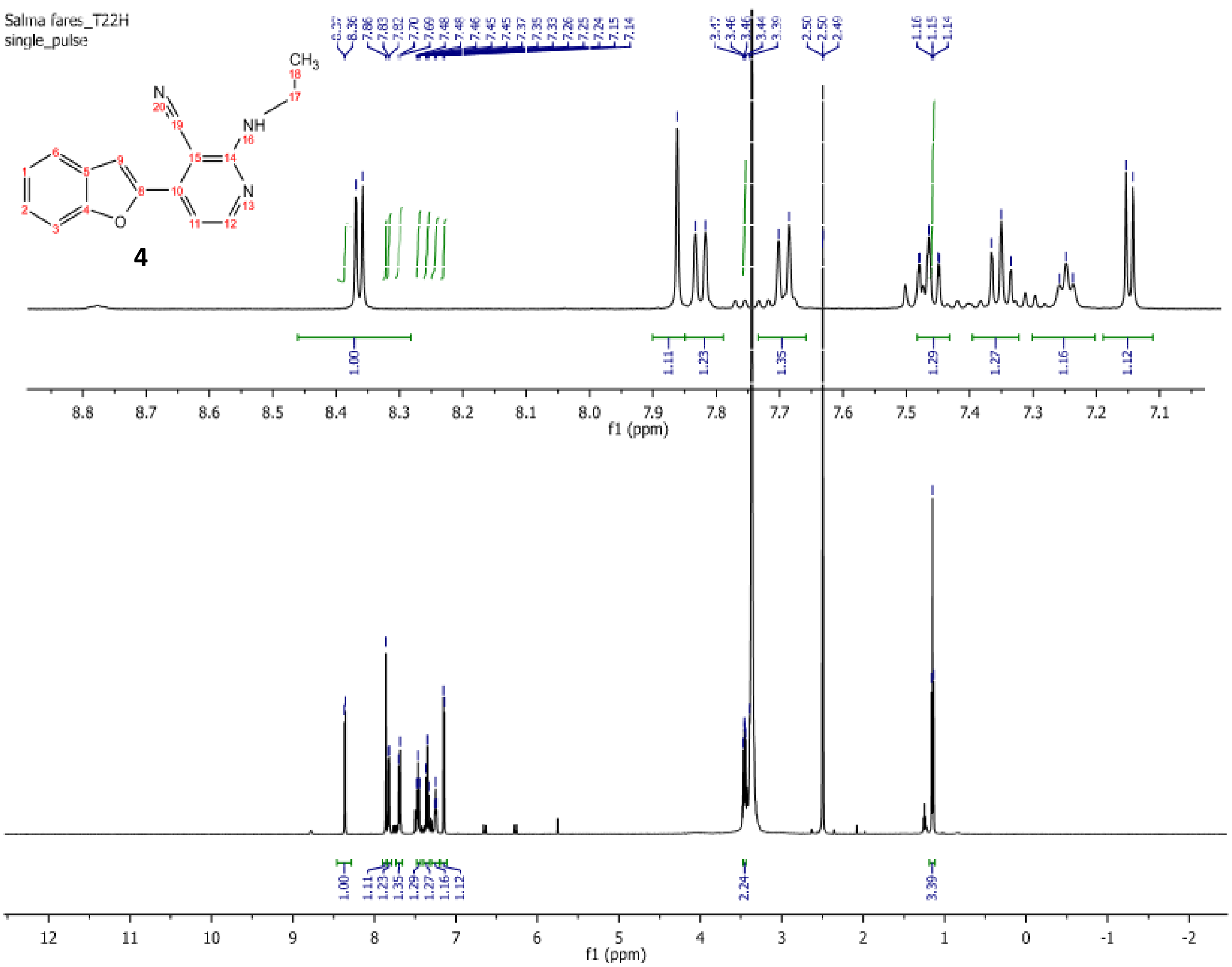

Chart 5. ^{1}H-NMR spectrum (500 MHz, DMSO-d_6) for compound **4**.

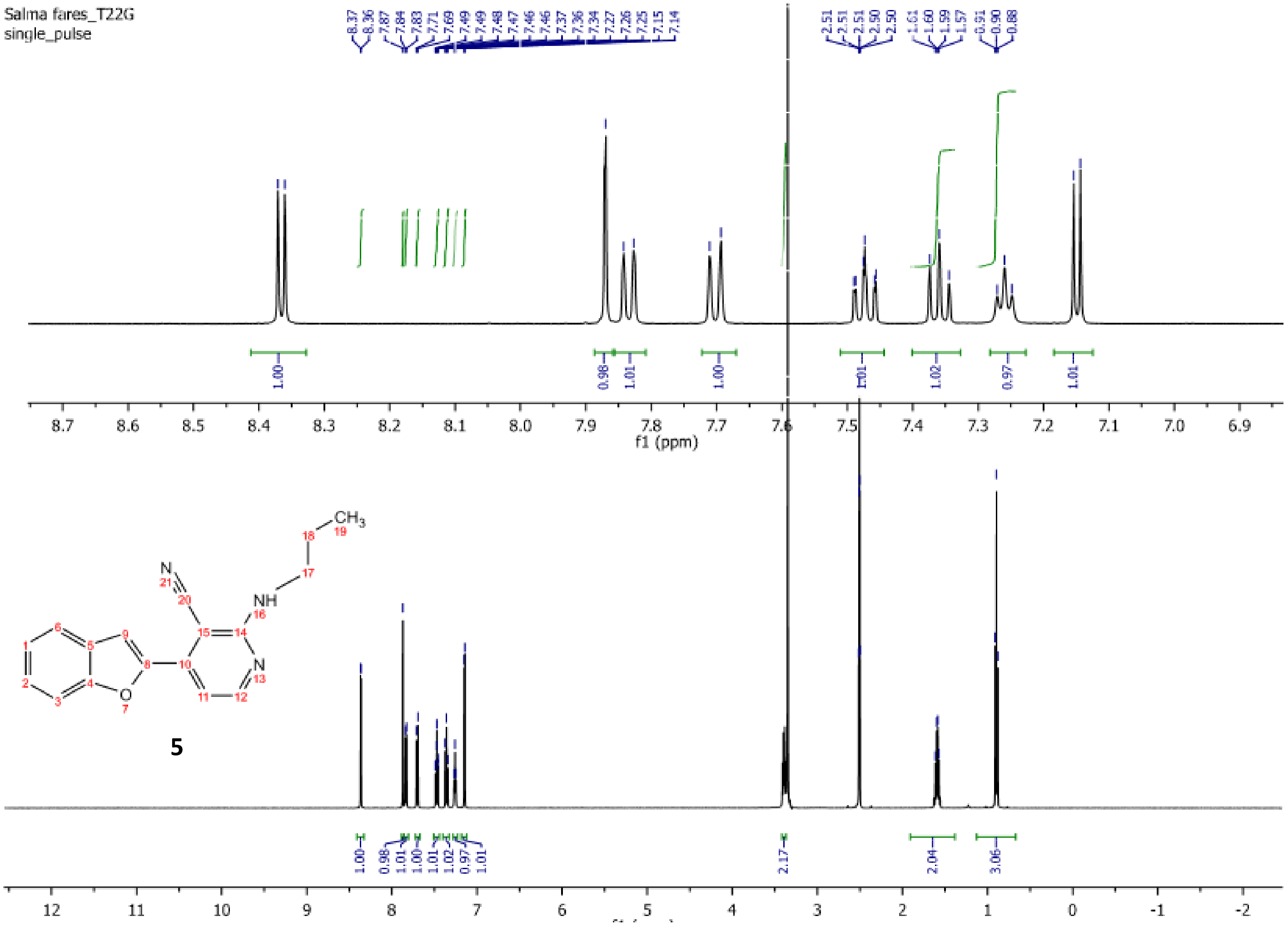

Chart 6. ^{1}H-NMR spectrum (500 MHz, DMSO-d_6) for compound **5**.

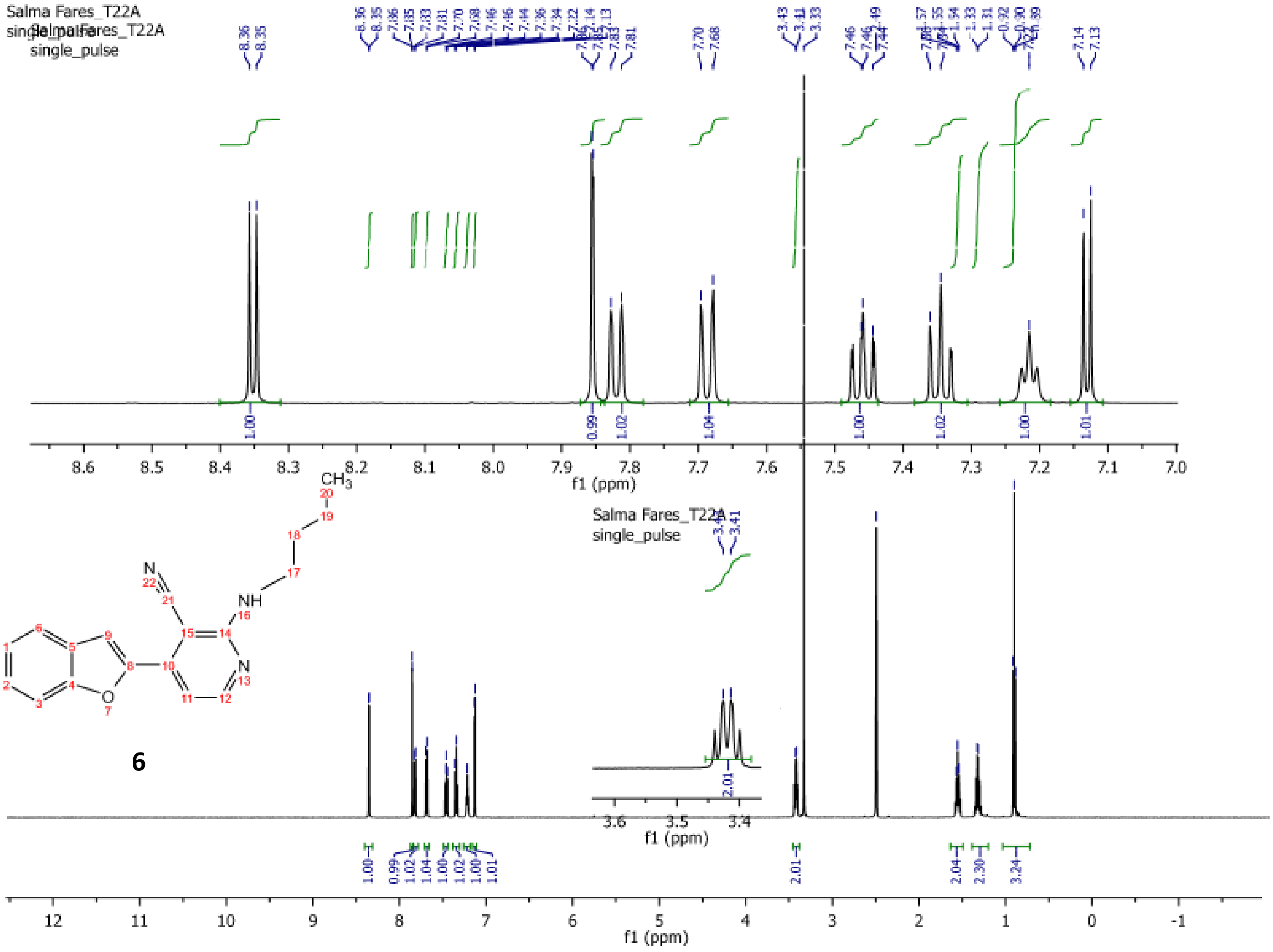

Chart 7. ^{1}H-NMR spectrum (500 MHz, DMSO-d_6) for compound **6**.

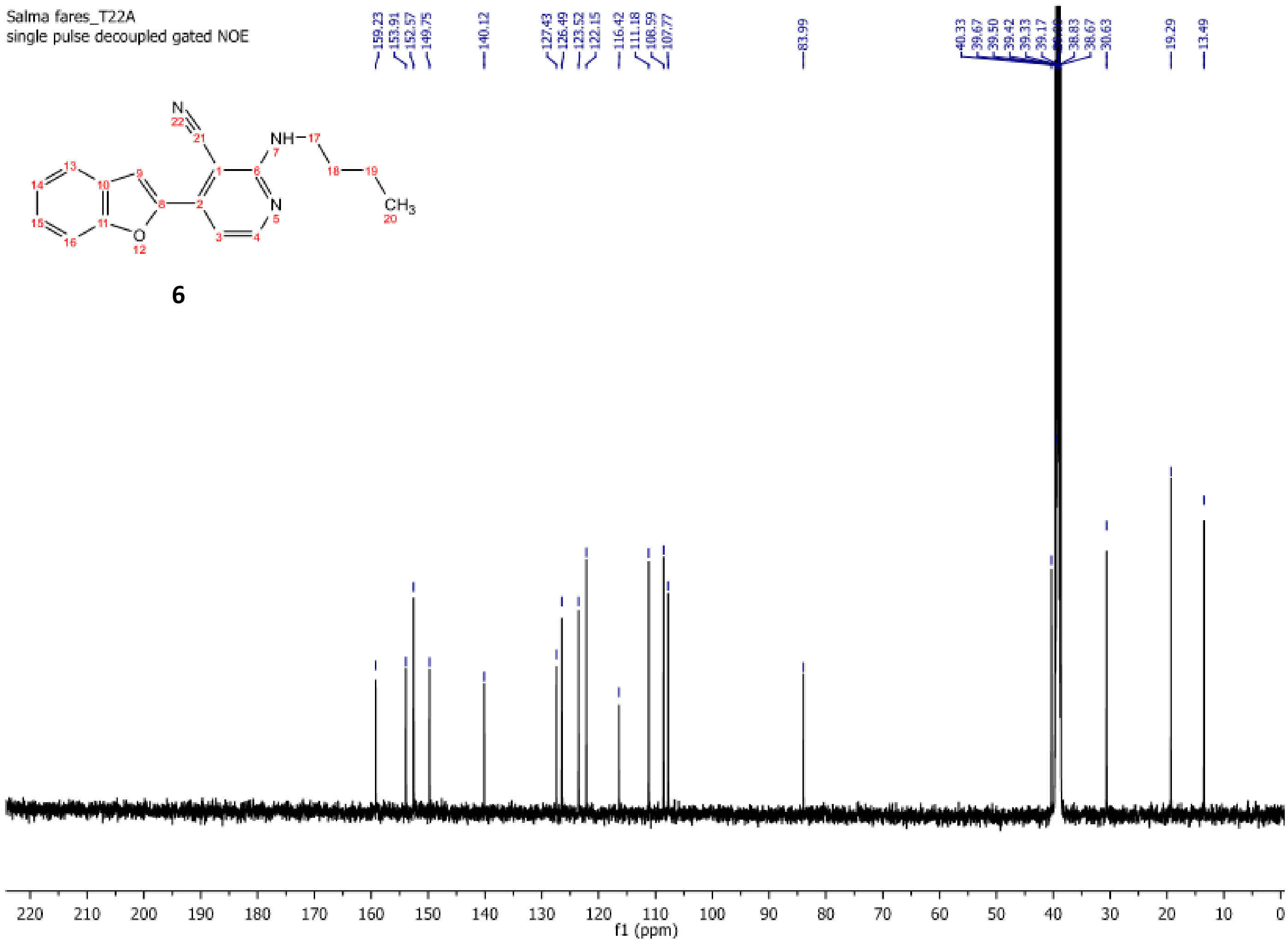

Chart 8. ^{13}C-NMR spectrum (125 MHz, DMSO-d_6) for compound **6**.

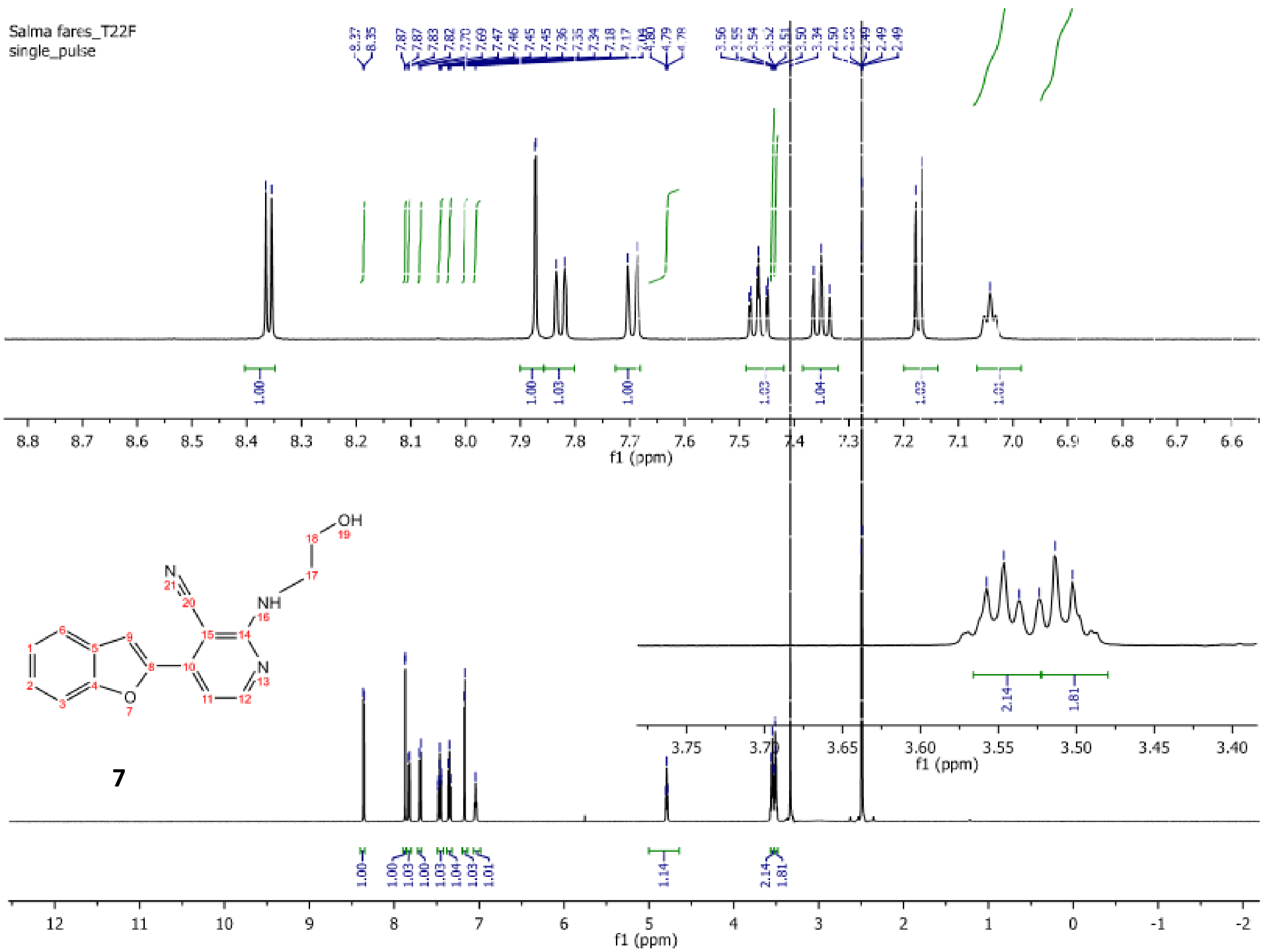

Chart 9. ^{1}H-NMR spectrum (500 MHz, DMSO-d_6) for compound **7**.

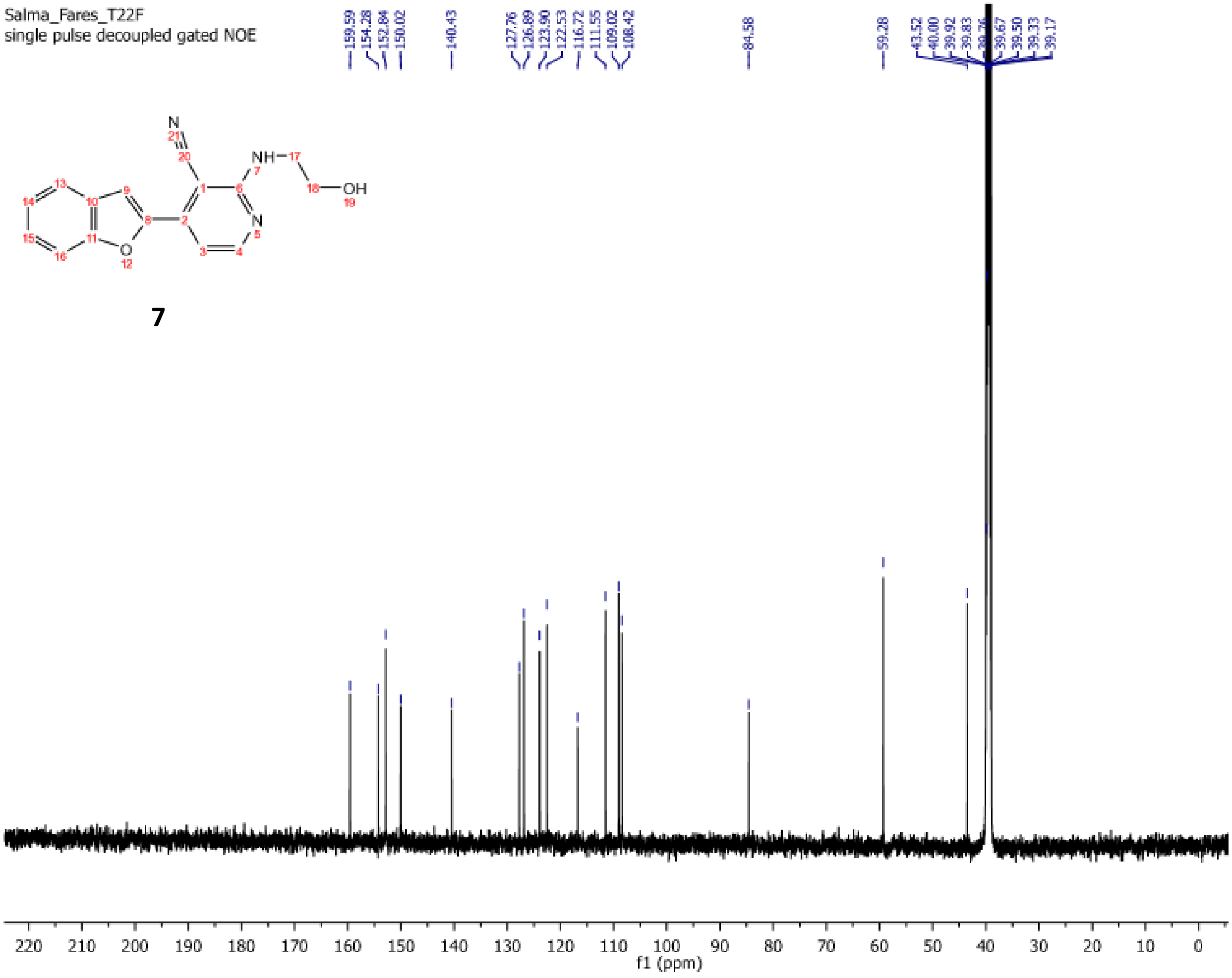

Chart 10. ^{13}C-NMR spectrum (125 MHz, DMSO-d_6) for compound **7**.

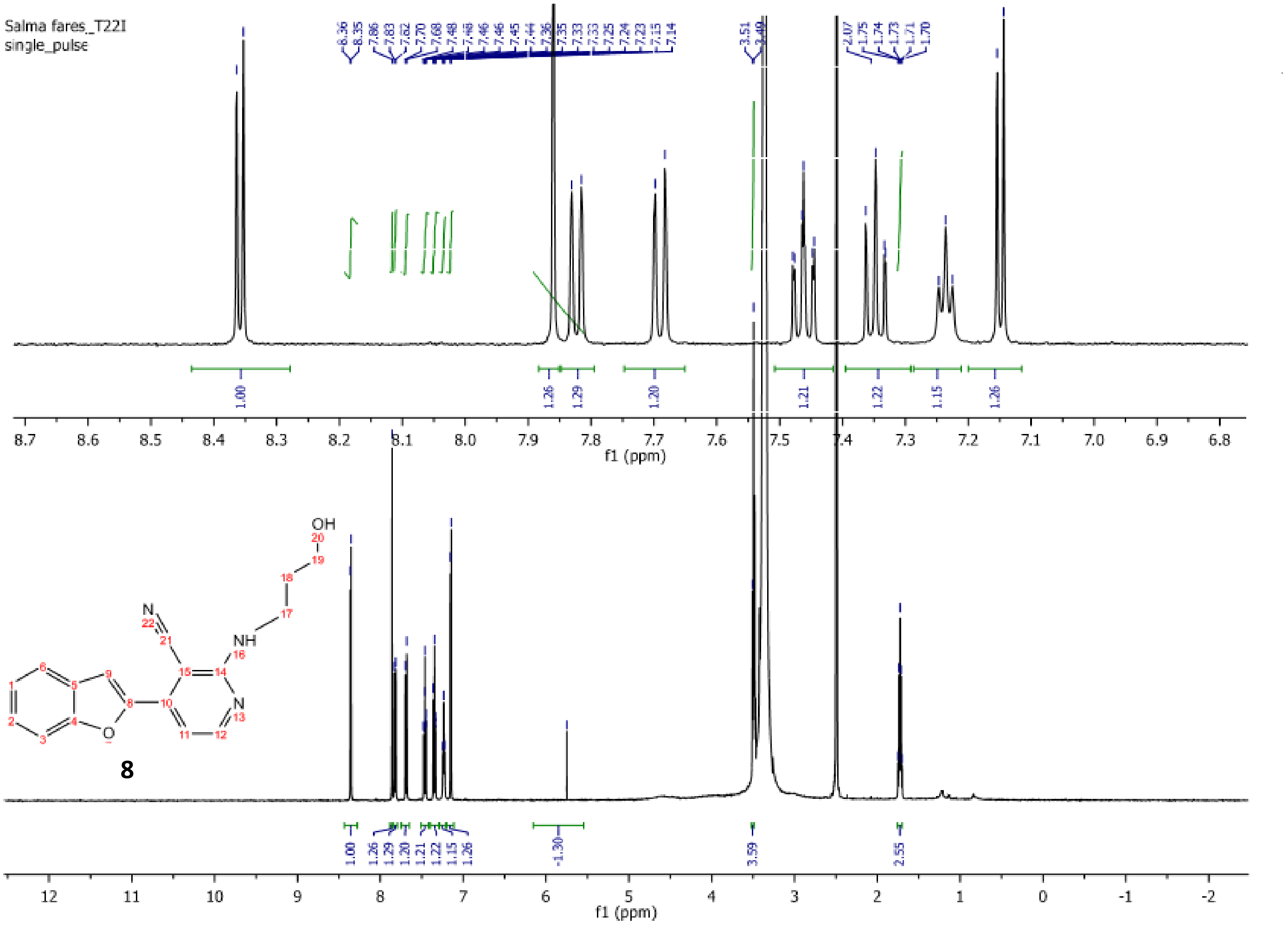

Chart 11. ^{1}H-NMR spectrum (500 MHz, DMSO-d_6) for compound **8**.

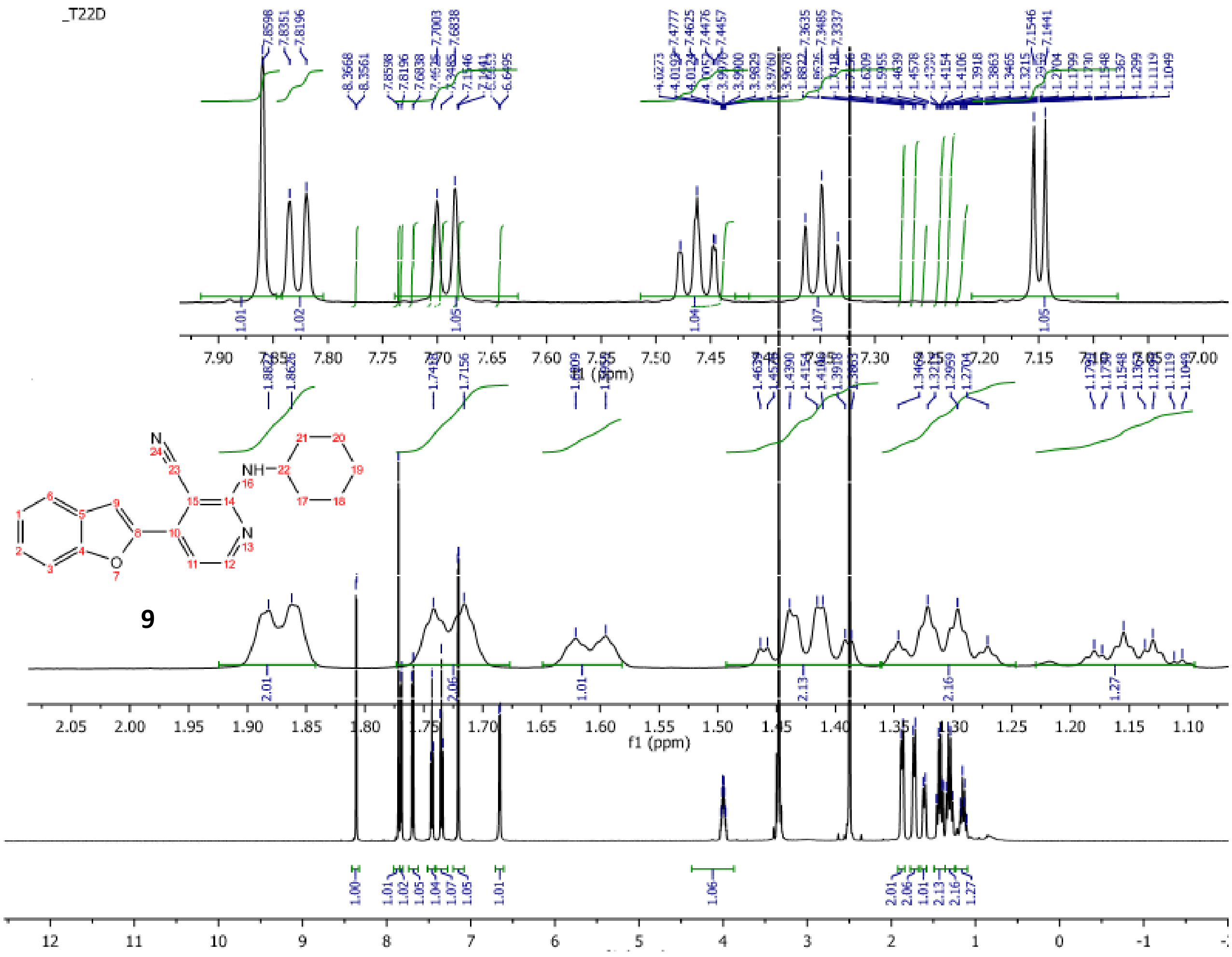

Chart 12. ^{1}H-NMR spectrum (500 MHz, DMSO-d$_6$) for compound **9**.

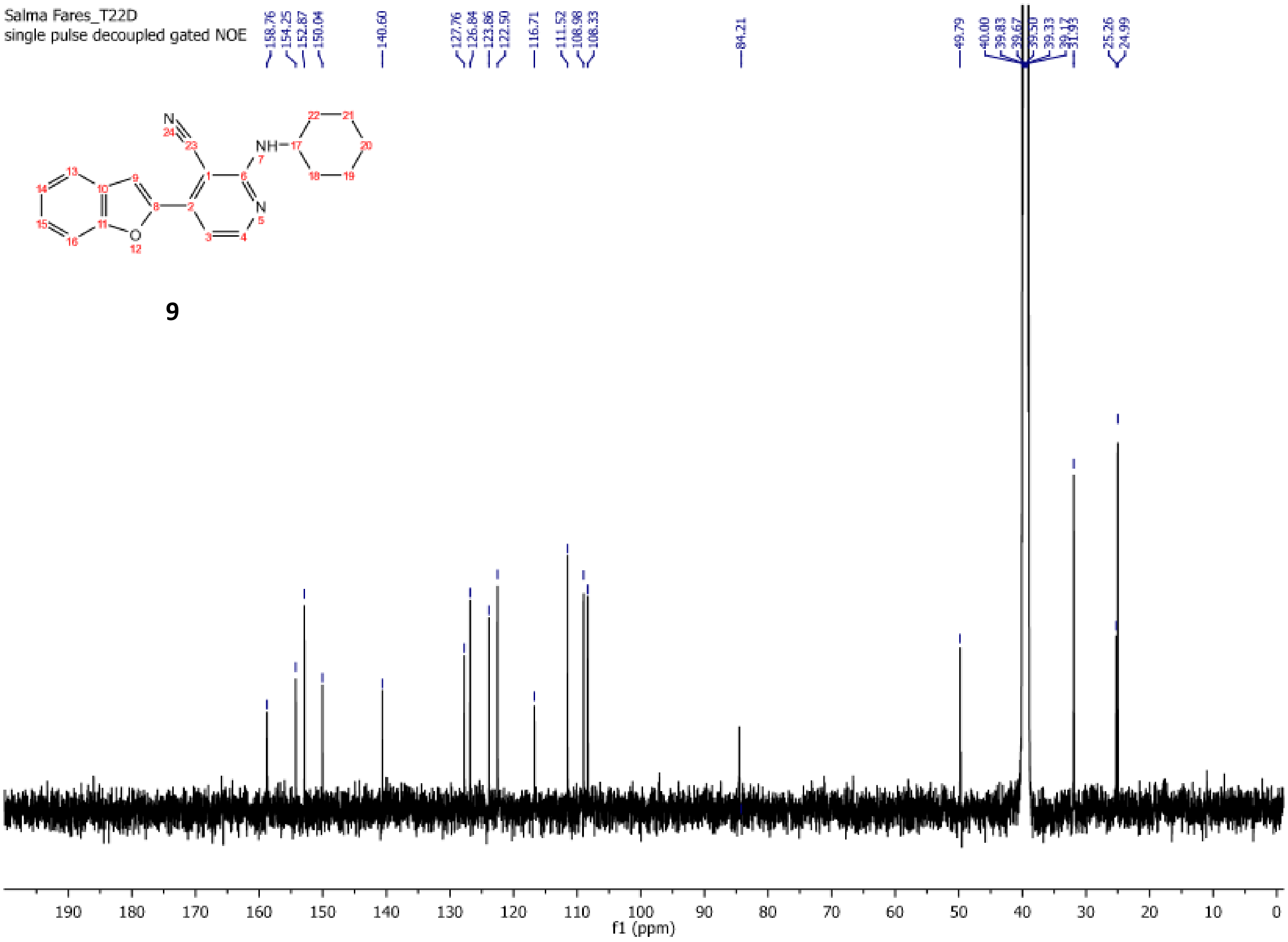

Chart 13. ^{13}C-NMR spectrum (125 MHz, DMSO-d_6) for compound **9**.

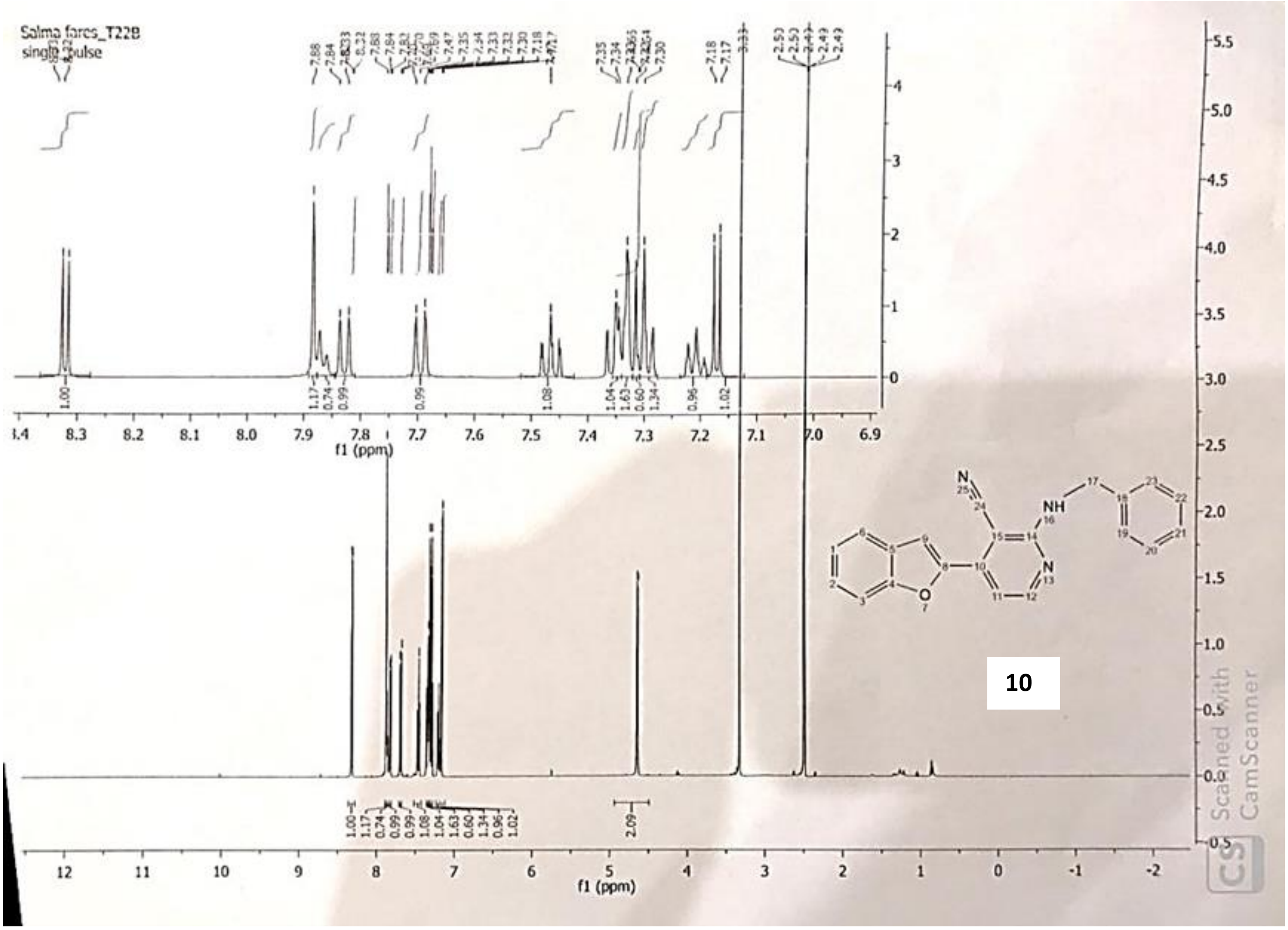

Chart 14. ^{1}H-NMR spectrum (500 MHz, DMSO-d_6) for compound **10**.

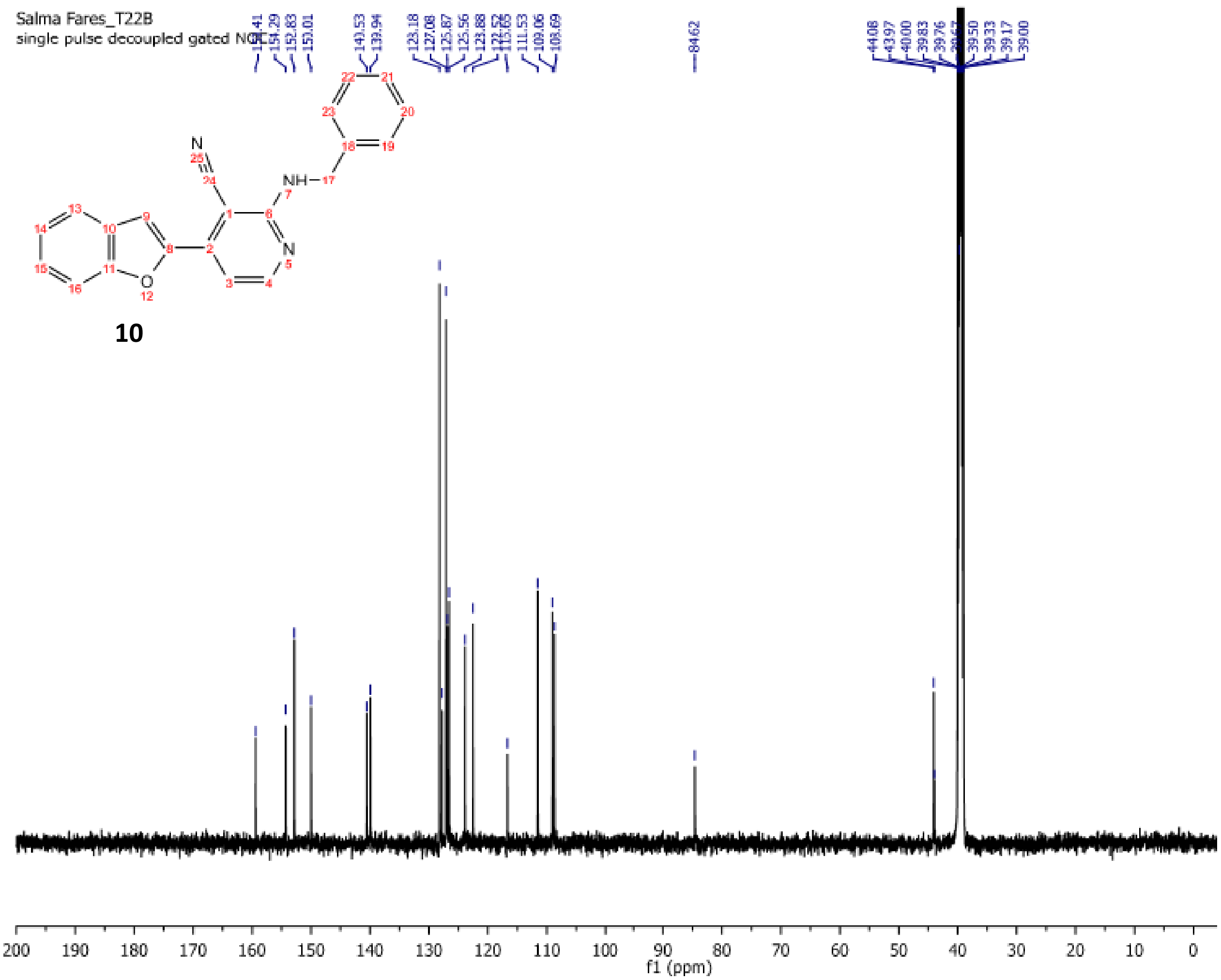

Chart 15. ^{13}C-NMR spectrum (125 MHz, DMSO-d_6) for compound **10**.

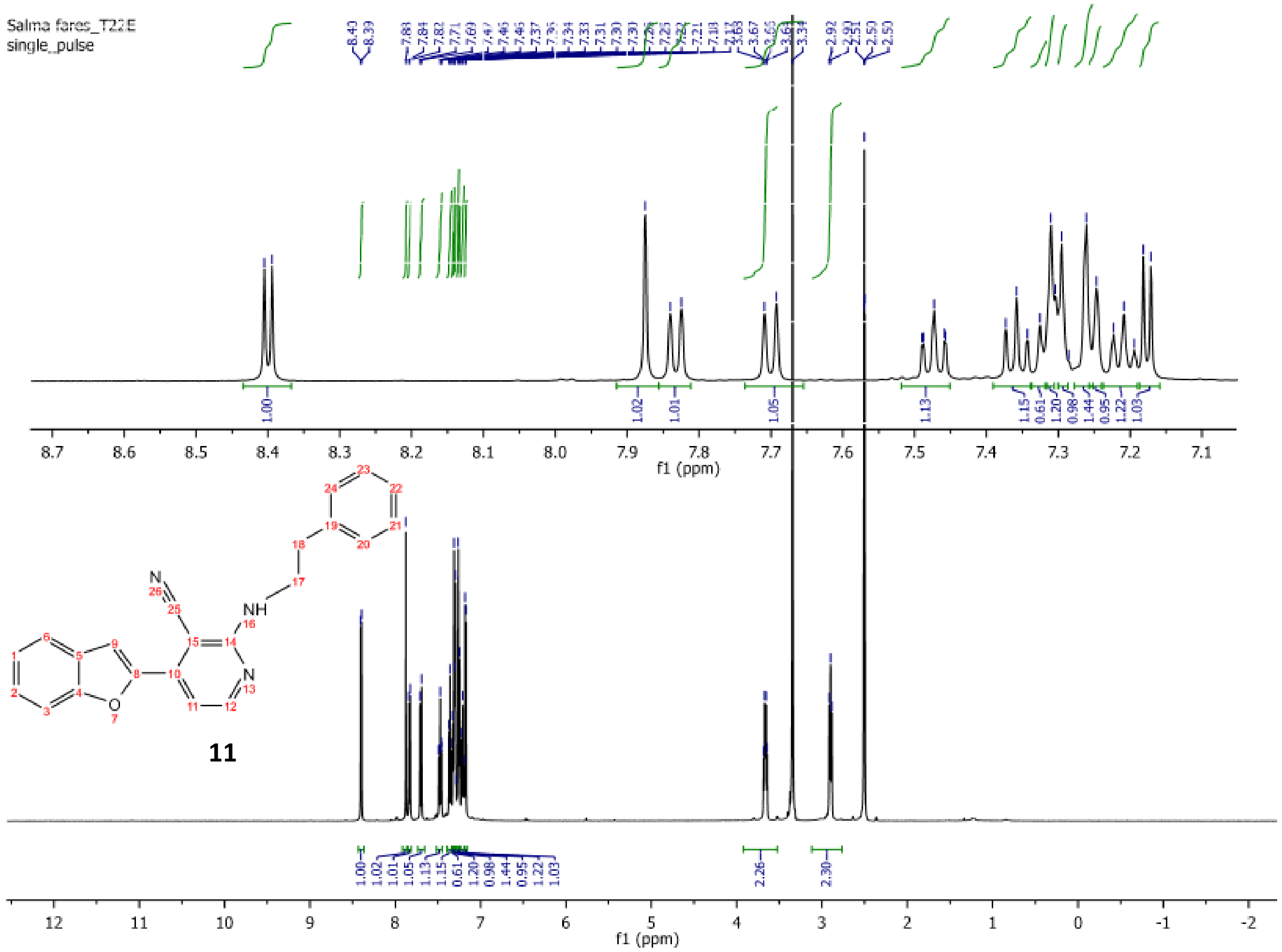

Chart 16. ^{1}H-NMR spectrum (500 MHz, DMSO-d_6) for compound **11**.

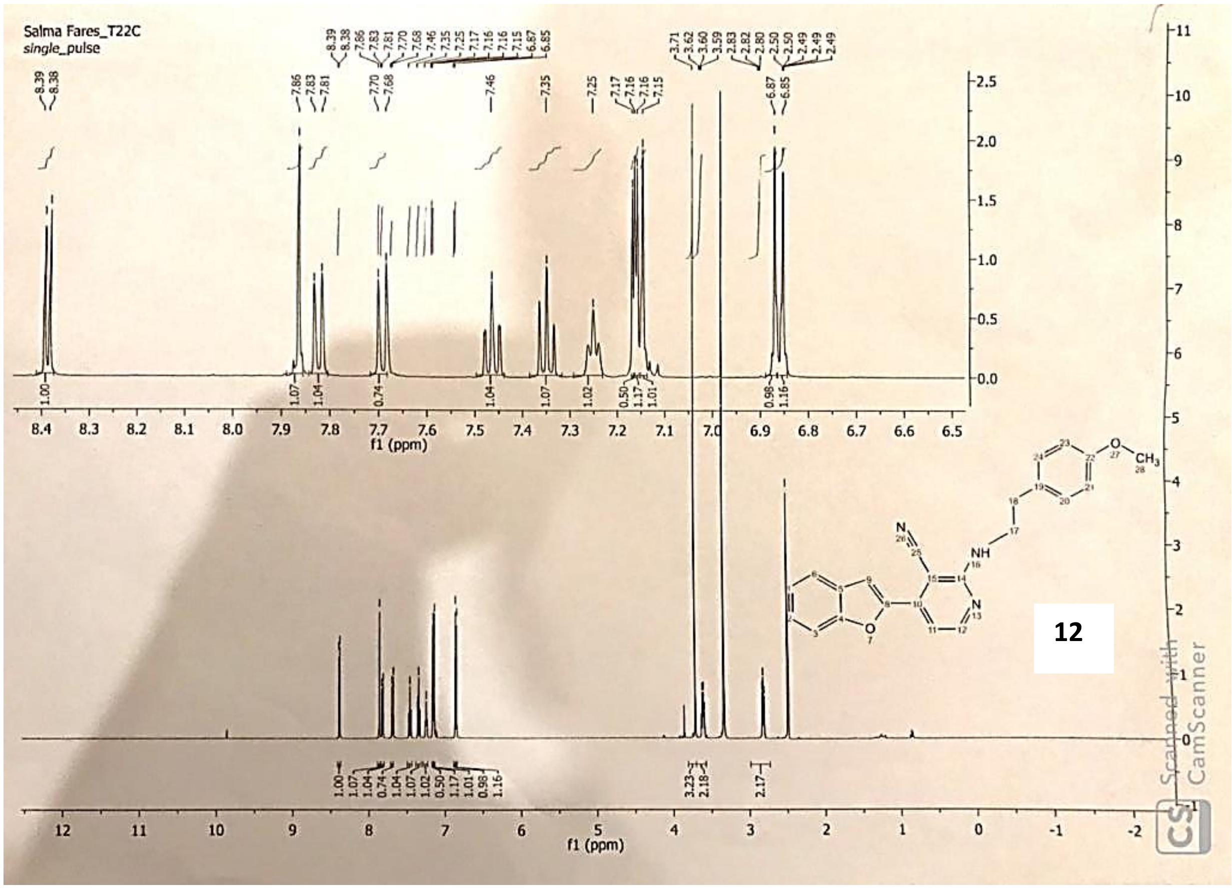

12

Chart 17. ^{1}H-NMR spectrum (500 MHz, DMSO-d_6) for compound **12**

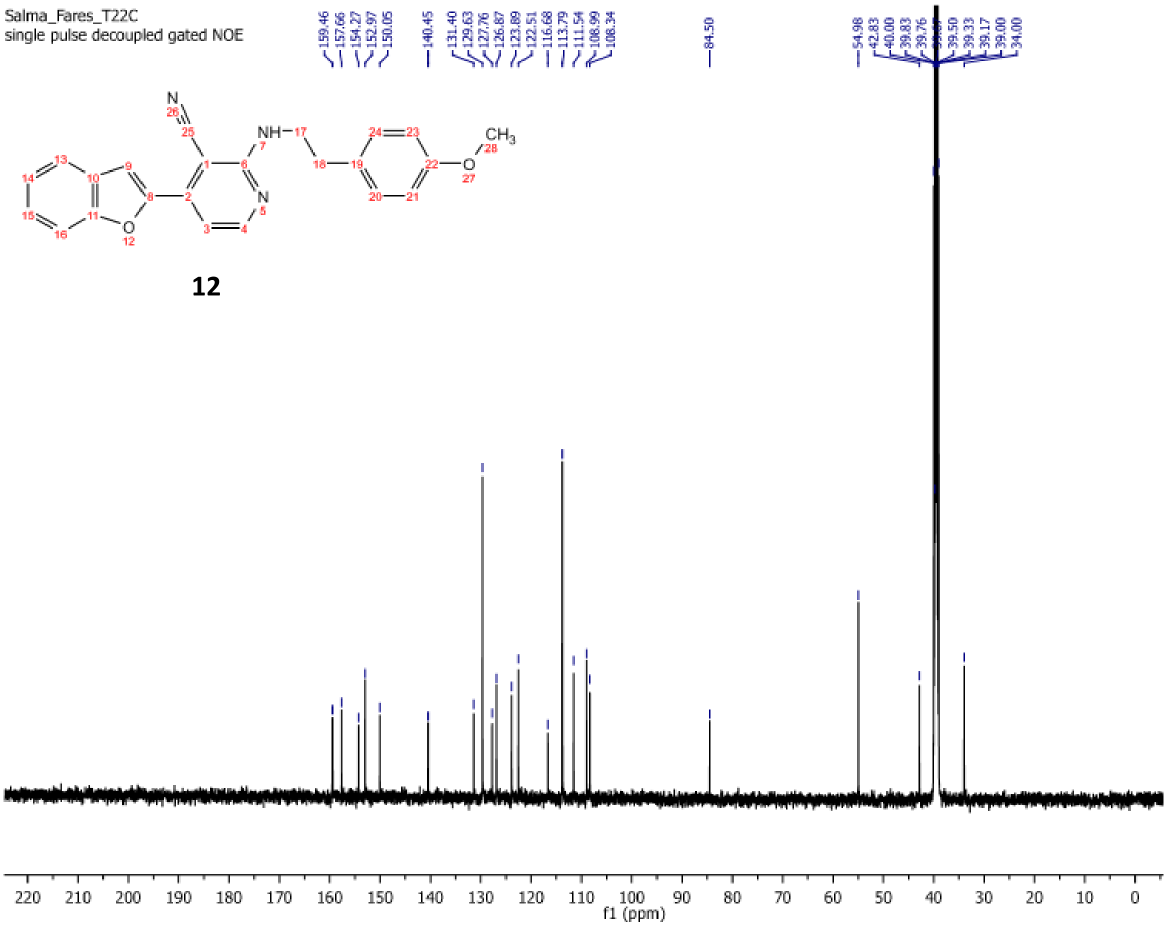

Chart 18. ^{13}C-NMR spectrum (125 MHz, DMSO-d_6) for compound **12**.

2. Experimental procedure

2.1. Antiproliferative screening

The *in vitro* antiproliferative activity of all synthesized compounds was evaluated by an MTT assay according to the reported method [47, 48] using five different human cancer cell lines, including hepatocellular carcinoma (HEPG-2), breast cancer (MCF-7), colorectal carcinoma (HCT-116), cervical carcinoma (HeLa), and human prostate cancer (PC-3). The cell lines were obtained from ATCC via a holding company for biological products and vaccines (VACSERA), Cairo, Egypt. The MTT assay is a colorimetric assay used for measuring the cellular growth based on the reduction of the yellow MTT to a purple formazan derivative by mitochondrial succinate dehydrogenase in viable cells. The color intensity of the formazan dye is correlated to the number of viable cells. The absorbance of colored solutions was measured employing a plate reader (EXL 800, USA) and the $IC_{50\%}$ values were calculated using dose response curves. The percentage of relative cell viability was calculated as (A570 of treated samples)/(A570 of untreated sample) × 100. The cytotoxic activity was defined as the concentration of the compound that caused 50% growth inhibition compared with the growth of an untreated cell.

2.2. Epidermal growth factor inhibition assay

In vitro luminescent EGFR tyrosine kinase assay, using Kinase-Glo® MAX as a detection reagent using DP-Glo™ reagent that measures the adenosine diphosphate (ADP) formed from a kinase reaction, were performed; this luminescent signal positively correlates with ADP amount and kinase activity. In 17 µl of distilled water, 6 µl of Kinase assay buffer and 1 µl ATP and 1 µl PTK substrate were mixed (master mixture). In every well, mix 20 µl of the master mixture and 5 µl of Inhibitor solution (Test Inhibitor) for positive control, and use 5 µl of the same solution without inhibitor (Inhibitor buffer) as the blank solution; add 20 µl of diluted EGFR enzyme and incubate at 30°C for 40

min. Add 50 µl of Kinase-Glo Max reagent to each well, cover the plate with aluminum foil and incubate the plate at room temperature for 15 min. Afterward, measure luminescence using the microplate reader. “Blank” value is subtracted from all readings. All samples and controls should be tested in triplicates.[36-38].

2.3. Caspase-3 assay

Sandwich enzyme-linked immunosorbent assay (ELISA) was used to determine the level of human active caspase-3 [49]. The tested compounds were used in 10 µM concentration. The tested compounds were carefully added to the cells and lysed using a cell extraction buffer. The cells were then incubated at room temperature for 4 h and then washed 4 times. Subsequently, 100 µL of an Invitrogen caspase-3 (active) monoclonal antibody solution was added and the cells were incubated again at room temperature for 2 h. The wells were accurately washed four times. Following the addition of 100 µL of a horseradish peroxidase (HRP)-labeled Anti-Rabbit IgG, the wells were incubated for 30 min at room temperature. The cells were washed prior to the addition of the chromogen. Finally, the absorbance was measured at 450 nm. The intensity of the resulting colored product was directly proportional to the concentration of human active protein in the original specimen.

2.4. Cell cycle analysis and induction of apoptosis

2.4.1. Flow cytometry analysis of the cell cycle distribution

For flow cytometry analysis of the DNA content, exponentially growing HCT-116 and MCF-7 cells were treated with compounds **3** and **11** at 10 µM concentration, respectively, for 24 h. After an incubation period, the cells were collected, centrifuged, and fixed with ice-cold ethanol (70%). The cells were subsequently treated with a buffer containing RNAse A and 0.1% Triton X-100, and stained

with PI. The cells were compared with the control, which was treated with DMSO. The DNA content was measured by flow cytometry [50-52].

2.4.2. Analysis of cellular apoptosis

Exponentially growing HCT-116 and MCF-7 cells were treated with compounds 3 and 11 at 10 μM concentration, respectively, and then incubated for 24 h. Following incubation, 1–5 × 10^5 cells were harvested and suspended in 500 μL of the binding buffer, 5 μL of annexin V-FITC, and 5 μL of PI. The cells were then incubated at room temperature for 5 min in the dark, and then at 37 °C. The annexin V-FITC conjugate is specially engineered to produce an enhanced fluorescence signal and photostability. The annexin V-FITC kit included annexin V-FITC for detecting apoptosis as well as PI for detecting necrosis. Thus, apoptosis and necrosis could be effectively differentiated. Detection and analysis of the annexin V-FITC binding by flow cytometry was conducted using a FITC signal detector (typically FL1). PI staining was evaluated by a phycoerythrin emission signal detector (usually FL2) [50-52].

2.3. Molecular docking study

The molecular modeling calculations and docking studies were performed using the MOE software version 2008.10 (Chemical Computing Group Inc., Montreal, Quebec, Canada) [61]. The X-ray crystallographic structure of EGFR with erlotinib was obtained from the RCSB protein data bank (PDB ID: 1m17). The energy was reduced using the MMFF94x force field. The conformers of the generated hybrids were docked into the EGFR active site using a triangle matcher placement method. Rescoring 1 was set up as London dG, while Affinity dG was chosen for rescoring 2. A molecular mechanics force field refinement was conducted on the top 100 produced poses. A validated docking protocol in the active site was subsequently used to examine the ligand–receptor interactions for the synthesized derivatives to predict their binding mode and binding affinity.
